# Active ingredients Isorhamnetin of Croci Srigma inhibit stomach adenocarcinomas progression by MAPK/mTOR signaling pathway

**DOI:** 10.1038/s41598-023-39627-z

**Published:** 2023-08-03

**Authors:** Xue-feng Shi, Qi Yu, Kai-bo Wang, Yi-dong Fu, Shun Zhang, Zhen-yun Liao, Yan Li, Ting Cai

**Affiliations:** 1 Department of Experimental Medical Science, Ningbo NO.2 Hospital, Ningbo, 315010 China; 2 Department of Pulmonary and Critical Care Medicine, Qinghai Provincial People’s Hospital, Xining, 81000 China; 3 Qinghai Red Cross Pioneer Search and Rescue Team, Xining, 810000 China; 4 Department of Oncology, Qinghai University Affiliated Hospital, Xining, 810001 Qinghai China

**Keywords:** Gastric cancer, Pharmacodynamics

## Abstract

Gastric cancer (GC) remains the third leading cause of cancer-related mortality in the world, and ninety-five percent of GC are stomach adenocarcinomas (STAD). The active ingredients of Croci Stigma, such as Isorhamnetin, Crocin, Crocetin and Kaempferol, all have antitumor activity. However, their chemical and pharmacological profiles remain to be elusive. In this study, network pharmacology was used to characterize the action mechanism of Croci Stigma. All compounds were obtained from the traditional Chinese medicine systems pharmacology (TCMSP) database, and active ingredients were selected by their oral bioavailability and drug-likeness index. The targets of Croci Stigma active ingredients were obtained from the traditional Chinese medicine integrated database (TCMID), whereas the related genes of STAD were obtained from DisGeNET platform. Cytoscape was used to undertake visual analyses of the Drug Ingredients–Gene Symbols–Disease (I–G–D) network, and 2 core genes including MAPK14, ERBB3 were obtained, which are the predicted targets of isorhamnetin (IH) and quercetin, respectively. Data analysis from TCGA platform showed that MAPK14 and ERBB3 all upregulated in STAD patients, but only the effect of MAPK14 expression on STAD patients’ survival was significant. Molecular docking showed that IH might affect the function of MAPK14 protein, and then the underlying action mechanisms of IH on STAD were experimentally validated using human gastric cancer cell line, HGC-27 cells. The results showed that IH can inhibit cell proliferation, migration, clonal formation, and arrest cell cycle, but promote the apoptosis of HGC-27 cells. qRT-PCR data demonstrated that IH downregulated the MAPK14 mRNA expression and EMT related genes. WB results showed that IH regulates MAPK/mTOR signaling pathway. These findings suggest that IH has the therapeutic potential for the treatment of STAD.

## Introduction

Gastric cancer (GC) is one of the fifth most common tumors and remains the third leading cause of cancer-related mortality in the world, affects more than one million people^1–4^. The 5-years s urvival rate of GC in United States is 31%, in United Kingdom is 19%, and in Europe is 26%^5^. A gastric carcinoma remains a burden worldwide as the prevalence of H. Pylori has not substantially decreased. Among the gastric carcinomas, stomach adenocarcinoma (STAD) are the most common type. However, there is still a lack of effective treatment for STAD.

Current cancer treatments for gastric cancer patients include surgical intervention, radiation, taking chemotherapeutic drugs and targeted therapeutic agents^6,7^. Most of patients with cancer have reached the advanced stage while diagnosed. 2 cytotoxic drugs are preferred as first-line systemic therapy for advanced gastric cancer patients because of their lower toxicity. First-line treatment with irinotecan-based regimens has been explored in clinical trials in advanced gastroesophageal cancers patients^8^. Moreover, several targeted therapeutic agents, including trastuzumab^9^, pembrolizumab/nivolumab^10,11^ and entrectinib/larotrectinib^12,13^, have been approved by the FDA for use in advanced gastric cancer. It is important to improve the quality of life and reduce pain for advanced stage patients without indications of surgery, radiotherapy, and chemotherapy. On this basis, traditional Chinese medicine monomers with multi-level, multi-target effects, high efficiency and low toxicity give full play to its advantages and benefit the overall treatment, so special attention must be focused to herbal medicine^14,15^.

Croci Stigma, a traditional Chinese medicine, is commonly used to activate blood circulation^16^. Its active ingredients, such as Isorhamnetin, Crocin, Crocetin and Kaempferol, have antitumor activity^17–21^. However, their underlying mechanism of antitumor remains elusive.

**Figure 1:**
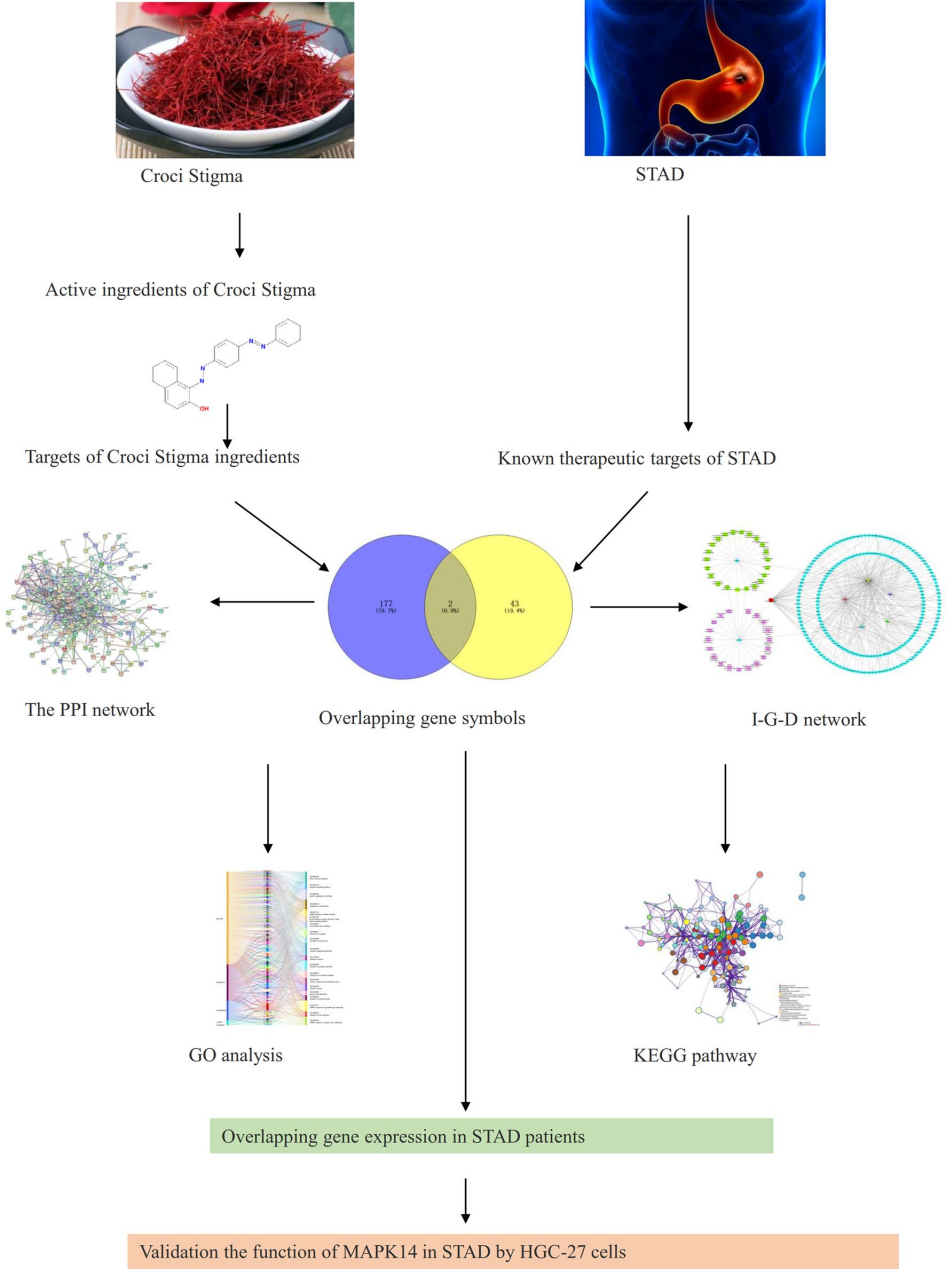
The workflow of this study.

This study applies network of pharmacology, molecular docking together with experiments to investigate the underlying mechanism of the active ingredients of Croci Stigma in antitumor activity for potential STAD treatment (Fig. 1).

## Materials and methods

### Active ingredients screening

Croci Stigma active ingredients were screened from TCMSP database (https://old.tcmsp-e.com/tcmsp.php) by Oral bioavailability (OB) ≥ 30%, and Drug-likeness (DL) ≥ 0.18.

### Prediction of targets for active ingredients of Croci Stigma and STAD

Active ingredient targets of Croci Stigma were predicted by TCMSP database. DisGeNET platform were used to screen STAD targets, which integrated data from the scientific literature, GWAS catalogues, expert curated repositories, and animal models.

### Screening of STAD-related genes targeted by the active ingredients of Croci Stigma

A Venn diagram was used to visualize the number of overlaps between STAD targets and Croci Stigma active ingredients by Venny2.1. The target genes of active ingredients and STAD, the overlapped genes, as well as overlapped genes by GO enrichment were visualized by Cytoscape software. Transcriptional regulatory relationships between transcription factors and related targets were obtained by TRRUST (https://www.grnpedia.org/trrust/).

### Construction of a drug ingredients–gene symbols–disease (I–G–D) network

After the active ingredients targets and STAD related genes were obtained, we built a network of complex information based on interactions between the Croci Stigma active ingredients, gene symbols, and disease (STAD), and used Cytoscape to undertake visual analyses of the I–G–D network (www.cytoscape.org/).

### Construction of protein–protein interaction networks (PPICN)

PPICN refers to the correlation between compounds and disease-related protein molecules, considering biochemistry, signal transduction, and genetic networks. The obtained intersection genes were uploaded onto STRING11.5.

### Enrichment of KEGG pathway, GO function, and Protein–Protein interaction (PPI)

KEGG pathway and Gene Ontoloty (GO) functional enrichment of the active ingredients target genes and STAD-related targets were enriched, as well as PPI, to explore their functions. Data and visulalized results were obtained by Metascape platform^22^.

### MAPK14 and ERBB3 expression and survival of STAD patients

The MAPK14 and ERBB3 expression, as the core genes of Croci Stigma active ingredients and STAD disease, and survival of STAD patients were obtained by UALCAN platform^23^ based on the Cancer Genome Atlas (TCGA) database, which provide easy access to publicly available cancer OMICS data (TCGA, MET500 and CPTAC). TP53 mutation status was obtained from TCGA whole exome sequencing data, the sample with/without TP53 mutation were matched with RNA-seq data.

### Cell lines and drug treatment

GES-1, HGC-27, and AGS cells was obtained from the Cell Bank of the Chinese Academy of Sciences, and stored in our Lab. GES-1, HGC-27 cells and AGS cells were cultured in RPMI-1640 medium (Invitrogen) supplemented with 10% fetal calf serum. Isorhamnetin (IH) was bought from MedChemExpress company, and dissolved in DMSO to prepare 10 mM storage solution, stored in – 80 °C. We treated HGC-27 with IH by 20 μM and 30 μM.

### RNA extraction and reverse transcriptase polymerase chain reaction (qRT-PCR)

Total RNA was extracted from HGC-27 cells by Total DNA/RNA Isolation Kit R6731 (Omega BIO-TEK, GA, U.S.A.) according to the manufacturer’s instructions. MAPK14 expression was detected by qRT-PCR. β-actin, sense: 5′-GCGGGAAATCGTGCGTGAC-3′ and antisense: 5′-GGAAGGAAGGCTGGAAGAG-3′. MAPK14, sense: 5′-ATTTCAGTCCATCATTCATGCG-3′ and antisense: 5′-GTAAAAACGTCCAACAGACCAA-3′.

### Western blotting

The HGC-27 cells treated by IH were lysis by lysis buffer with 1%PMSF and 1% phosphatase inhibitors, then protein concentration was measured by BCA protein assay kit (Thermo Fisher Scientific, IL, U.S.A.). GAPDH (Cell Signaling Technology, MA, U.S.A.), MAPK14 (Santa Cruz Biotechnology, Inc., Texas, U.S.A.), Caspase-3 (Cell Signaling Technology, MA, U.S.A.), p-mTOR (Cell Signaling Technology, MA, U.S.A.), and mTOR (Cell Signaling Technology, MA, U.S.A.) protein expression was detected by western blotting according our previous study^24^.

### Cell proliferation with RTCA

HGC-27 cells and AGS cells proliferation in real-time were performed by the xCELLigence Real-Time Cell Analysis (RTCA) DP instrument (Roche Diagnostics GmbH, Mannheim, Germany) at 37 °C with 5% CO_2_. HGC-27 cells were seeded on gold microelectrodes embedded at the bottom of 16 well microplates (E-plates; Roche Diagnostics, Basel, Switzerland) at a density of 1 × 10^4^ cells/well. The impedance was recorded at 15 min intervals. 20, 30, 40 and 60 µM of IH were added to the culture 28 h subsequent to seeding. All incubations were performed at a volume of 200 µl between 0 and 72 h.

### CCK8 proliferation assay

GES-1cells and HGC-27 cells were seeded into 96-well plate at 1 × 10^4^ cells/well in 100 μl 10%FBS RIPM-1640 medium. After discarding supernatant of each well, 100 μl RIPM-1640 containing 10%CCK-8 solution was added into wells after cultured for periods as indicated. The plates were placed into the incubator for 1.5 h, and detected with a microplate reader.

### Apoptosis assay

A total of 2.5 × 10^5^ GES-1, HGC-27 and AGS cells were seeded in 6-well plates to grow overnight. Then the medium containing 10%FBS RIPM-1640 was changed to 1%FBS RIPM-1640 medium with or without IH. Cells were harvested at 24 h and 48 h, and washed with PBS twice to analysis the degree of apoptosis using FITC Annexin-V Apoptosis detection kit according manufacturer’s instructions (BD Biosciences company, NJ, USA).

### Wound healing assay

HGC-27 cells with a density of 1 × 10^6^ cells/dish were cultured until monolayer cells were formed in 60 mm dishes. A straight line was drawn corssing dishes on the back of dishes using a maker pen. At least 5 lines passed through each dish. The “1” zigzag scratches were made on monolayer cultured cells using sterilized 100 μl yellow tip to form an acellular growth area. The relative distance of the scratch area was recorded. The dropped cells by scratching were then washed off with PBS, then medium containing 1%FBS was added to dishes with or without IH. After culturing for 0 h and 72 h, the relative distance of cell migration from the wound area was measured under the inverted microscope.

### Cell colony formation assay

HGC-27 cells and AGS cells were seeded in 6-well plates at a density of 120 cells per well to grow overnight. At the second day, change supernatant to 1%FBS RIPM-1640 with or without IH, and change medium every 3 days. After about 2 weeks, fixed cells with 10% paraformaldehyde at room temperature (RT) for 30 min and stained with 0.25% of crystal violet at RT for 30 min. Finally, the newly formed colonies were imaged and counted by ImageJ software.

### Cell cycle analysis

The cells were seeded in 6-well plates and treated by IH for 24 h, and the cell suspension was collected for cell cycle analysis. Cells were washed with PBS three times, the cell cycle was detected by using cell cycle staining kit (LianKe bio, Hangzhou, China). The experiment was repeated three times.

### Statistical analysis

All values are presented as the mean ± SD. Comparisons between two groups were analyzed via Student’s-tests. Differences between groups were considered to be significant at *P* < 0.05.

## Results

### Active ingredients of Croci Stigma

Aided by TCMSP database, total five Croci Stigma active ingredients were screened by Oral bioavailability (OB) ≥ 30%, and DL ≥ 0.18, showed in Supplementary Table 1.

### Prediction Identification of targets for active ingredients of Croci Stigma and KEGG pathway and GO-based functional enrichment of targets

**Figure 2:**
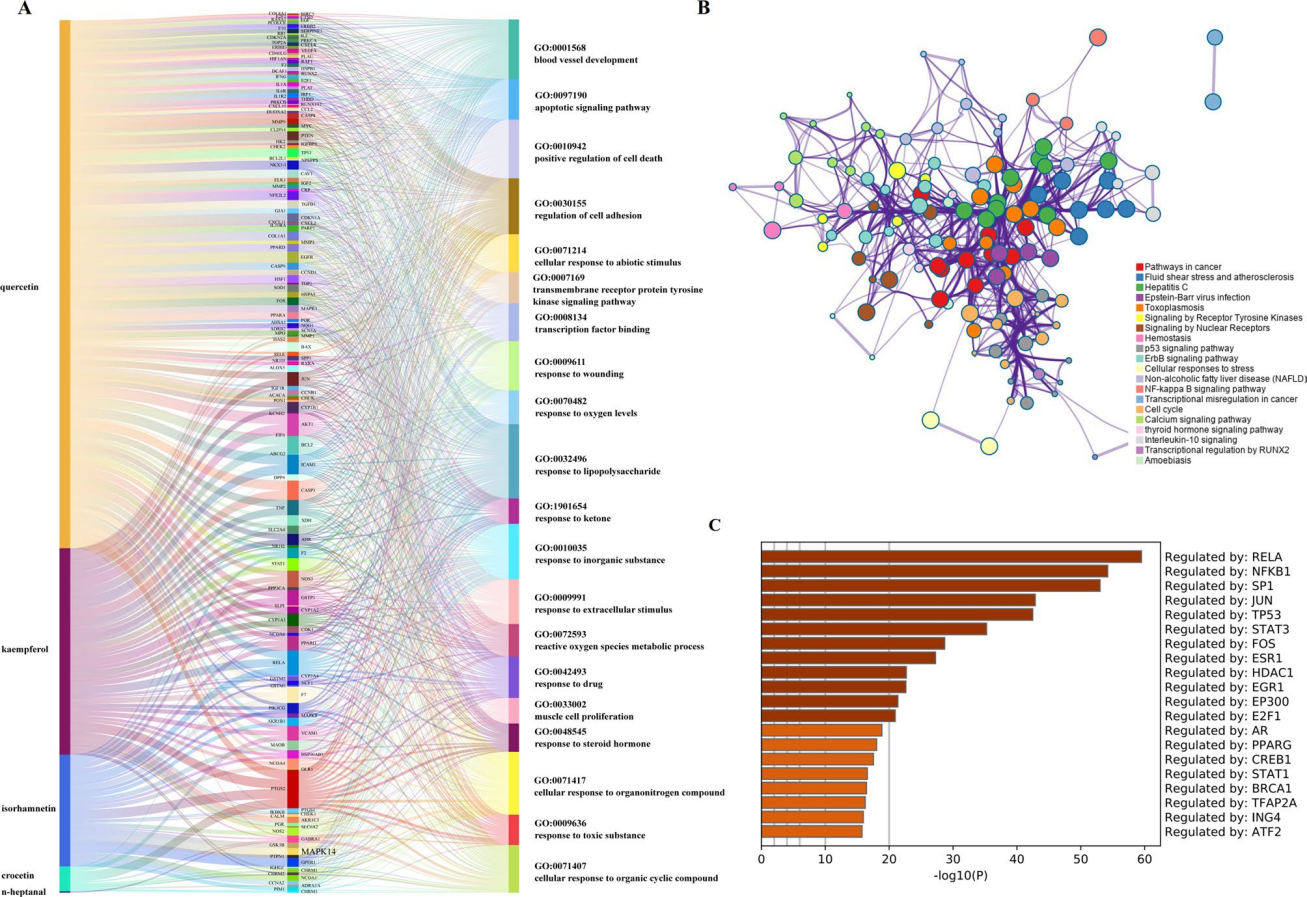
Function and target analysis of active ingredients of Croci Stigma. (**A**) Active ingredients and targets of Croci Stigma analyzed by Go enrichment analysis. (**B**) The transcription factors regulating active ingredient targets. (**C**) The transcription factor regulating active ingredient targets.

Five active ingredients of Croci Stigma and 188 related targets were obtained and showed in Fig. 2A. The left line is the Croci Stigma active ingredients, the middle line is the related targets of Croci Stigma active compounds, and the right line shows the related targets by GO functional enrichment. The GO enrichment showed the active compounds of Croci Stigma mainly enriched in apoptotic signaling pathway, regulation of cell adhesion, transcription factor binding, response to oxygen levels, response to extracellular stimulus, reactive oxygen species metabolic process, which are all associated with cancer progression. KEGG pathway showed Croci Stigma active compounds enriched in pathway in cancer, signaling by receptor tyrosine kinase, signaling by nuclear receptors, P53 signaling pathway, ErbB signaling pathway, NF-Kappa B signaling pathway, cell cycle, interleukin-10 signaling, and transcriptional regulation by RNUX2, which are also related to cancer progression (Fig. 2B). As showed in Fig. 2C, the active ingredient related targets can be regulated by transcription factor such as RELA, NFKB1, SP1, JUN, TP53, STAT3, FOS, ESR1, HDAC1, HDAC1, EGR1. PPI diagram (Extended data Fig. S1) showed that there was complicated interaction between all target protein of Croci Stigma active ingredients with Interaction Score ≥ 0.9. As showed in Supplementary Table 2, MAPK14 may interact with AKT1, CASP3, CD40LG, CXCL8, ELK1, FOS, HSPB1, IL-2, JUN, MAPK1, MYC, NCF1, RB1, RELA, STAT1, TNF, TP53, and VEGFA. While ERBB3 only interact with AKT1, EGF, EGFR, ERBB2, and MAPK8.

**Figure 3:**
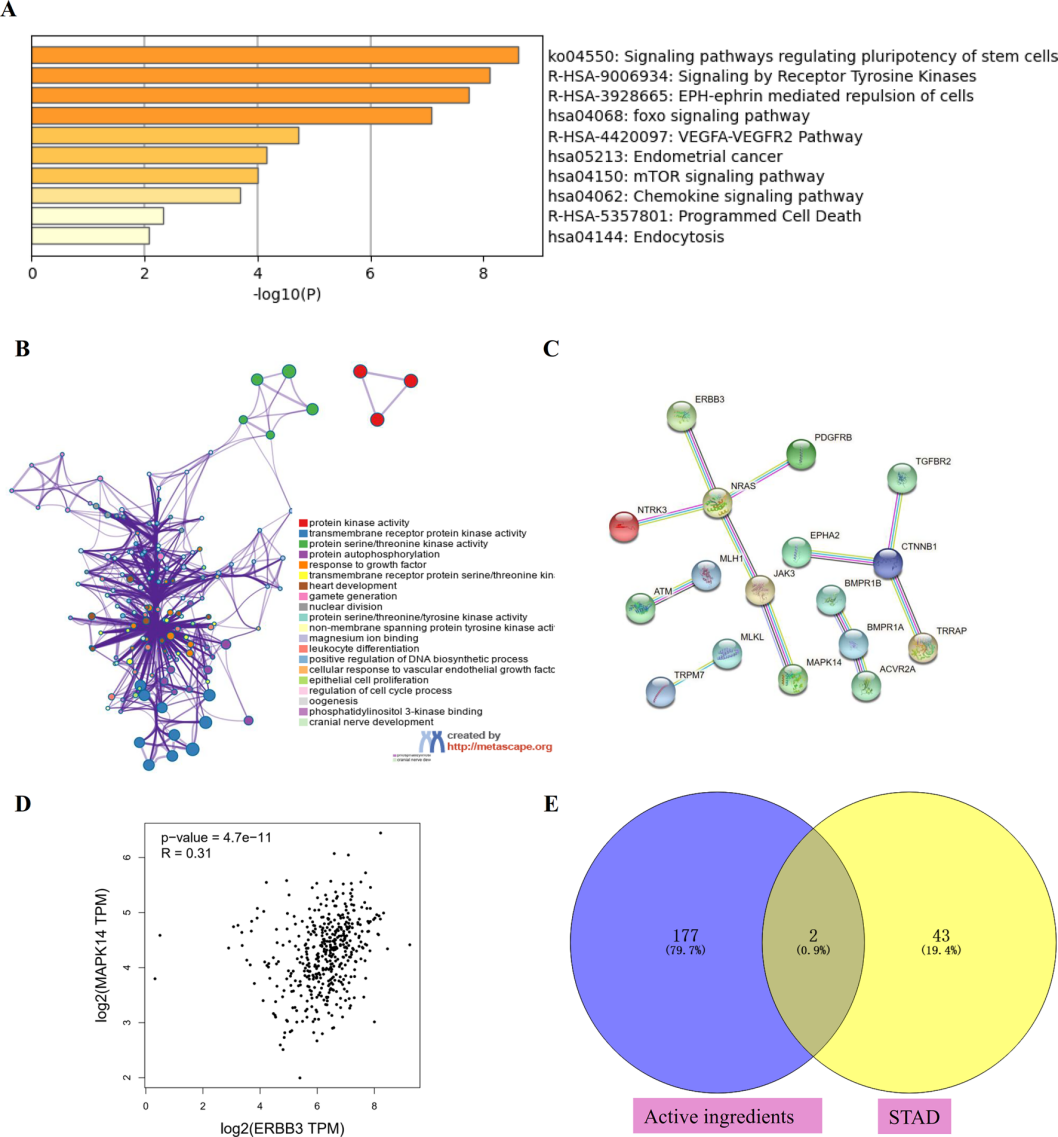
STAD related target genes and functional analysis. (**A**) KEGG pathway of related target genes of STAD^25^. (**B**) Go enrichment analysis of related target genes of STAD. (**C**) PPI of related target genes of STAD. (**D**) The correlation of 2 core genes in STAD patients by GEPIA2 platform. (**E**) core genes between active ingredients of Croci Stigma and STAD-Venn diagram.

45 STAD-related genes were screened by DisGeNET platform, and KEGG pathway enrichment of these genes mainly enriched in foxo signaling pathway, VEGFA-VEGFR2 pathway, mTOR signaling pathway, and programmed cell death (Fig. 3A). Moreover, these related genes enriched in protein kinase activity, transmembrane receptor protein kinase activity, protein autophosphorylation, response to growth factor (Fig. 3B). We also analysis the protein interaction of STAD targets by STING11.5 with interaction score > 0.9, and result showed that selected core genes (MAPK14, ERBB3) had indirect correlation in STAD (Fig. 3C). The correlation of these 2 core genes were also validated in STAD patients by GEPIA2 platform (Fig. 3D).

### Core action genes in Croci Stigma formulas and STAD disease

Moreover, the genes related to active ingredients of Croci Stigma were obtained, and a total of 188 target genes were included (Extended data Fig. S2, blue diamond). STAD (Synonym: stomach cancer, adenocacinoma; CUI:C0278701;) related targets were screened based on DisGeNET platform by score ≥ 0.3, and a total of 43 genes were involved (Extended data Fig. S2, blue diamond). There were 2 core genes (MAPK14 and ERBB3) between active ingredients of Croci Stigma and STAD, showed in Fig. 3E, Extended data Fig. 1 (blue diamond). MAPK14 is the related gene of IH, and ERBB3 is the related gene of quercetin. Go enrichment of the two core genes by KOBAS platform showed both genes enriched in positive regulation of gene expression, signaling transduction, and ATP binding, detail showed in Extended data Fig. 2 (green square).

### Results of molecular docking

Molecular docking showed that Croci Stigma active compound IH had a significant role in regulation of MAPK14 protein. IH formed 3 hydrogen bonds with the amino acid residues Leu108, Met109, and Asp168 in MAPK14, making IH and MAPK14 to form a stable complex (Extend Fig. S3A,B) with binding energy of − 7.230.

### Association with MAPK14 and ERBB3 expression and clinicopathologic variables in STAD patients

**Figure 4:**
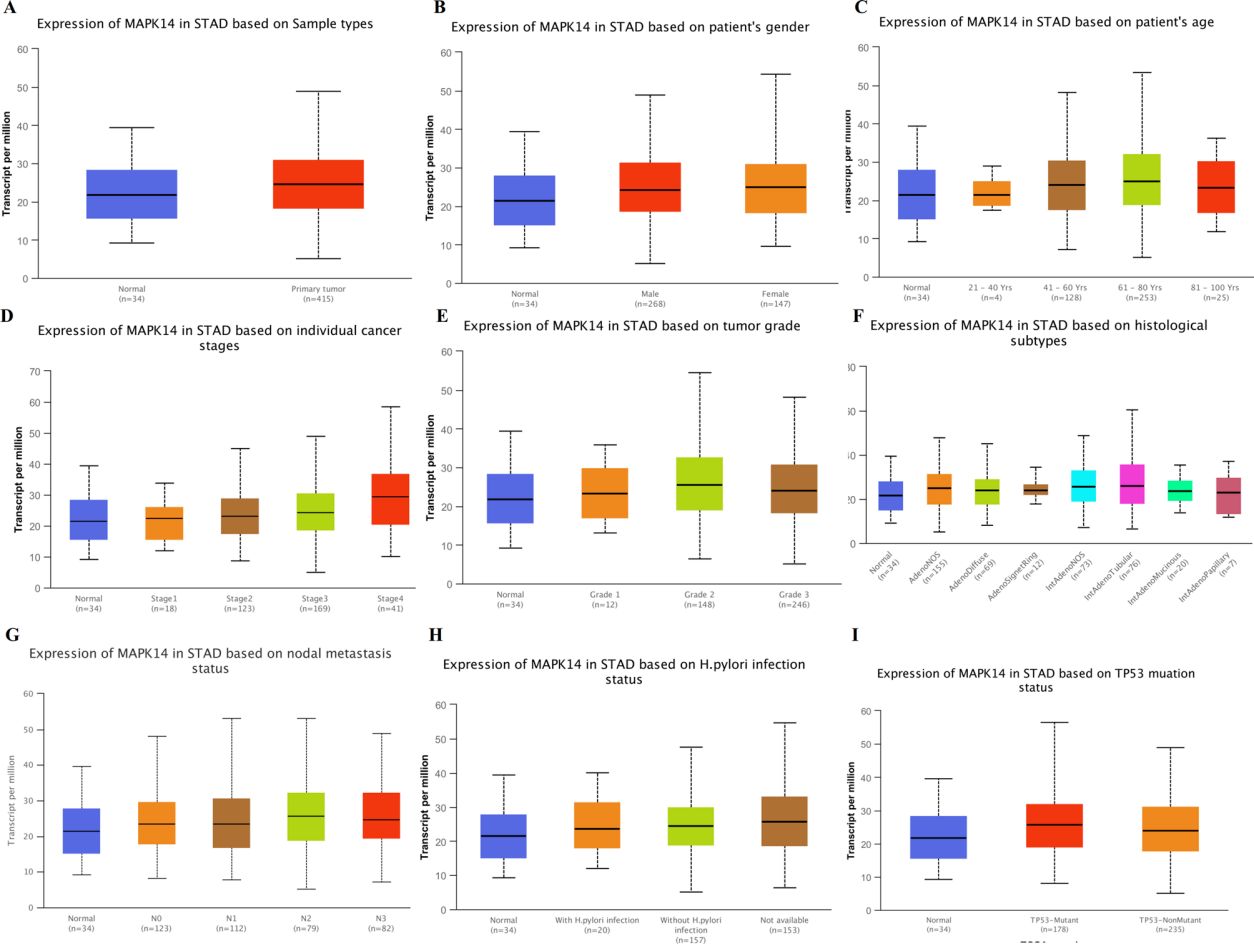
MAPK14 expression in STAD patients based on patients’ characteristics. (**A**) MAPK14 expression in total STAD. (**B**) MAPK14 expression in STAD based on patient’s gender. (**C**) MAPK14 expression in STAD based on patient’s age. (**D**) MAPK14 expression in STAD based on individual cancer stages. (**E**) MAPK14 expression in STAD based on tumor grade. (**F**) MAPK14 expression in STAD based on histological subtypes. (**G**) MAPK14 expression in STAD based on nodal metastasis status. (**H**) MAPK14 expression in STAD based on H. pylori infection status. (**I**) MAPK14 expression in STAD based on TP53 mutation status.

Based on the TCGA database, we plotted a box diagram of MAPK14 and ERBB3 expression for tumor tissues and adjacent normal samples. We found that MAPK14 was highly expressed in 5 of 24 tumor tissues (STAD, CHOL, ESCA, HNSC and LIHC) (Extended data Fig. S4A), and ERBB3 is highly expressed in 10 of 24 tumor tissues including STAD (Extended data Fig. S4B). A total of 34 normal tissues and 415 STAD samples were included from TCGA to analysis the survival probability of high and low/medium expression of MAPK14 and ERBB3 in STAD patients. And the results showed that low/medium MAPK14 expression had a better survival probability than high MAPK14 expression of STAD patients (Extended data Fig. S4C), while ERBB3 expression did not show significant effect on survival probability of STAD patients (Extended data Fig. S4D). Therefore, we only analyzed the expression of MAPK14 based on all patients’ characteristics. As showed in Fig. 4A, MAPK14 expression was upregulated in STAD tumor samples relative to normal tissues (*P* < 0.05). But, there was no significantly difference in MAPK14 expression in SATD patients between male and female (Fig. 4B). However, the MAPK14 expression in 61–80 years old patients with STAD (n = 253) was significantly higher than that in 81-100Yrs patients (n = 25), *P* < 0.05 (Fig. 4C). Moreover, MAPK14 expression increased gradually with the progress of STAD, MAPK14 expression in Stage3 and Stage4 STAD tumor tissues was significantly higher than that in normal samples, and MAPK14 expression in Stage4 STAD tumor tissues was remarkably higher than that in stage2 STAD tumor tissues,* P* < 0.05 (Fig. 4D). Moreover, there were significantly differential expression of MAPK14 between Grade2 STAD tumor tissues and normal sample, and between Grade1 and Grade 3 STAD tumor tissues (Fig. 4E). Based on histological subtypes, expression of MAPK14 was higher in AdenoNOS and IntAdenoNOS than that in normal samples, while MAPK14 expression in IntAdenoNOS was higher than that in AdenoDiffuse and IntAdenoMucinous (Fig. 4F). Subtype descriptions and pathologic N descriptions showed in Supplementary Table 3. As showed in Fig. 4G, there was no differences in MAPK14 expression among all pathologic lymph node metastasis period of STAD patients. To analysis the effect of H.pylori infection status on MAPK14 progression, all STAD patients divided to 3 groups, with H.pylori infection group, without H.pylori infection group, and unkown H.pylori infection status (not available) group. The MAPK14 expression increased in without H.pylori infection group and not available group compared to that in normal samples (Fig. 4H). Our results also showed that MAPK14 expression in STAD patients with TP53 mutation status was higher than that in normal samples, but not in STAD Patients without TP-53 mutation (Fig. 4I).

### IH regulates proliferation and survival of HGC-27 and AGS cells, but GES-1 cells

**Figure 5:**
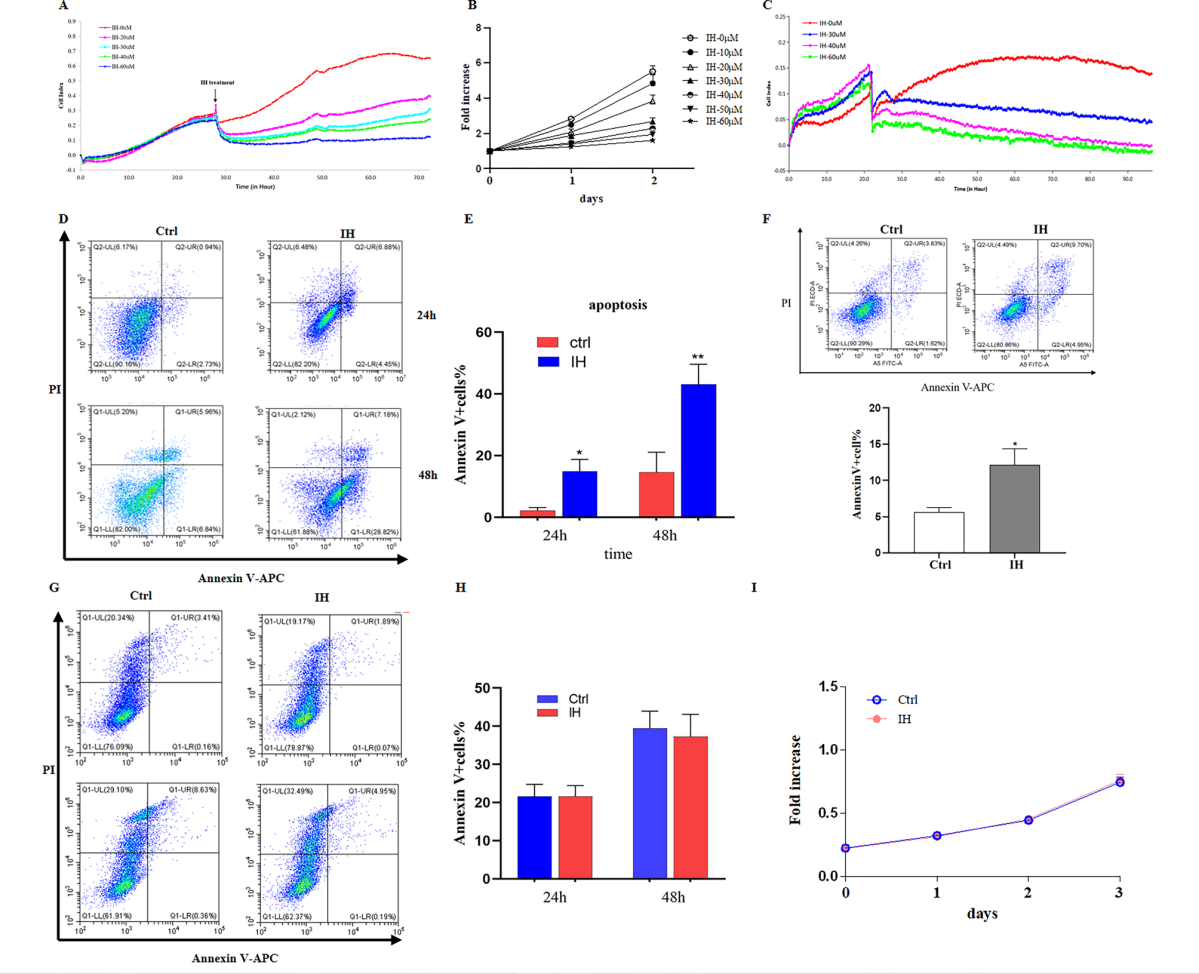
IH inhibits the proliferation and survival of HGC-27 cells. (**A**) Proliferation of IH-treated HGC-27 cells examined by RTCA. (**B**) Proliferation of IH-treated HGC-27 cells examined by CCK8. (**C**) Proliferation of IH-treated AGS cells examined by RTCA. (**D**) Apoptosis of IH-treated HGC-27 cells assayed by Annexin-V/APC staining. (**E**) Percentage of Annexin V + cells in IH-treated HGC-27 cells. (**F**) Apoptosis of IH-treated AGS cells assayed by Annexin-V/APC staining (up); Percentage of Annexin V + cells in IH-treated AGS cells (down). (**G**) Apoptosis of IH-treated GES-1 cells assayed by Annexin-V/APC staining. (**H**) Percentage of Annexin V + cells in IH-treated GES-1 cells. (**I**) Proliferation of IH-treated GES-1 cells examined by CCK8. Values are mean ± SD, n = 3, *P < 0.05, **P < 0.01 vs. control.

To verify the mechanism of IH’s treatment on STAD, we treated gastric cancer cells lines HGC-27 cells and AGS cells with 0-60 μM of IH. RTCA results showed that IH significantly inhibited HGC-27 and AGS cells proliferation in a dose-dependent manner (Fig. 5A,C), and showed obvious toxicity to HGC-27 cells and AGS cells when IH concentration was increased to 40 μM and 60 μM. Consistently, our CCK8 results also showed the similar trend in HGC-27 cells (Fig. 5B). To verify whether IH contributes to the apoptosis of HGC-27 and AGS cells, we treated HGC-27 and AGS cells with 30 μM IH. The results showed that IH significantly promoted the apoptosis of HGC-27 after coculture for 24 h and 48 h (Fig. 5D,E), and promoted the apoptosis of AGS cells treated with IH for 24 h (Fig. 5F), the percentage of Annexin-v + cells in IH treated HGC-27 and AGS cells was significantly increased. We also detected the toxicity of IH in gastric normal cell line GES-1 cells, we measured the proliferation and survival of GES-1 cells treated with 30 μM IH. We found that there is no obviously difference between apoptosis of GES-1 cells treated with or without IH (Fig. 5G,H). Moreover, IH did not have toxic in proliferation of GES-1 cells at different IH treatment time (Fig. 5I).

### IH regulates migration, colony formation, and cell cycle of HGC-27 and AGS cells

**Figure 6:**
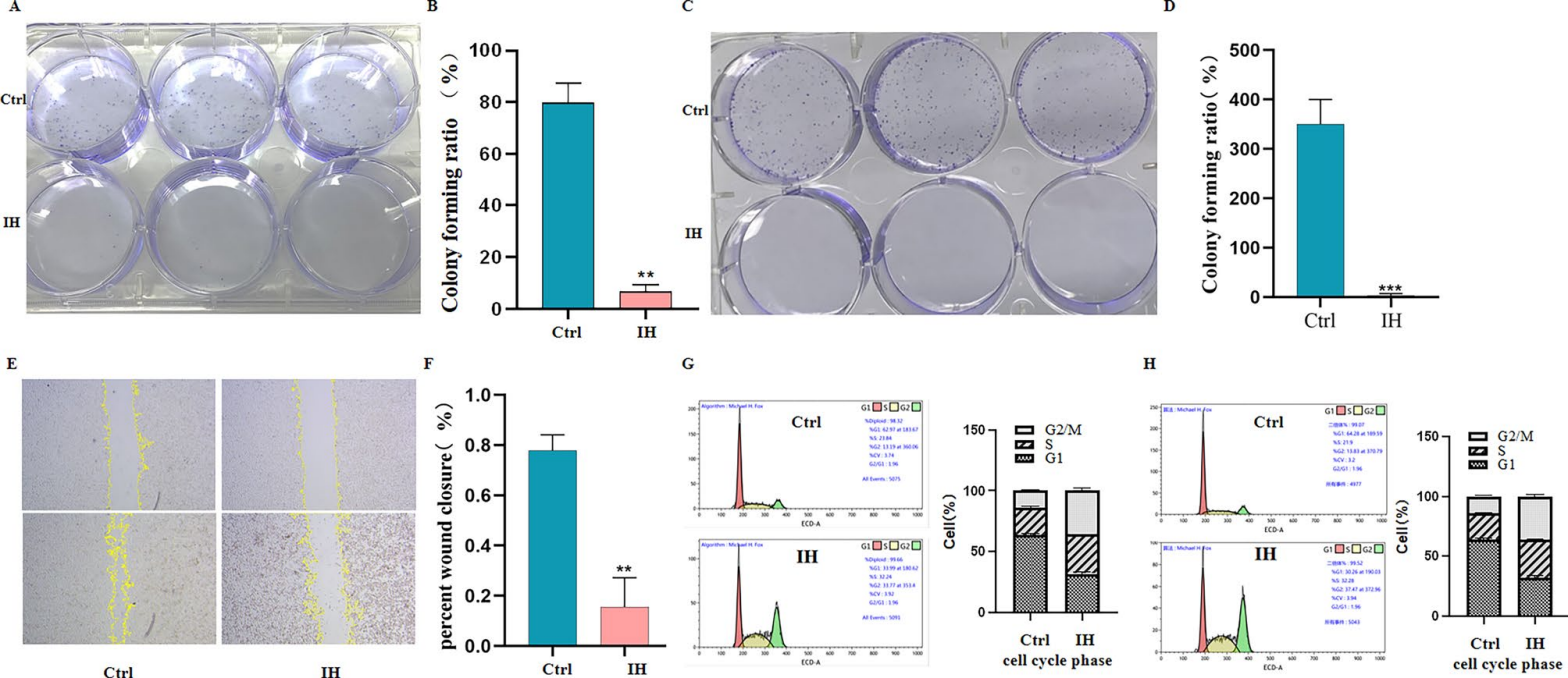
IH inhibits the colony formation, migration of HGC-27 and AGS cells. (**A**) Colony formation of IH-treated HGC-27 cells detected by clone formation assay. (**B**) Colony forming ratio in IH-treated HGC-27 cells. (**C**) Colony formation of IH-treated AGS cells detected by clone formation assay. (**D**) Colony forming ratio in IH-treated AGS cells. (**E**) Migration of IH-treated HGC-27 cells. (**F**) Percent of wound closure (%) of HGC-27 cells. (**G**) Cell cycle of IH-treated HGC-27 cells (left); Cell cycle phase of IH-treated HGC-27 cells (right). (**H**) Cell cycle of IH-treated AGS cells (left); Cell cycle phase of IH-treated AGS cells (right). Values are mean ± SD, n = 3, *P < 0.05, **P < 0.01 vs. control.

Next, we detected the effect of IH on HGC-27 and AGS cell colony formation, and the results showed that 30 μM IH obviously inhibited clone formation of HGC-27 cells and AGS cells (Fig. 6A–D). Our wound healing assay results indicated that IH markedly inhibited the migration of HGC-27 cells (Fig. 6E,F). Meanwhile, more cells were found to be arrested in G2/M phase in HGC-27 cells and AGS cells as well (Fig. 6G,H).

### IH regulates epithelial-mesenchymal transition of HGC-27 cells

**Figure 7:**
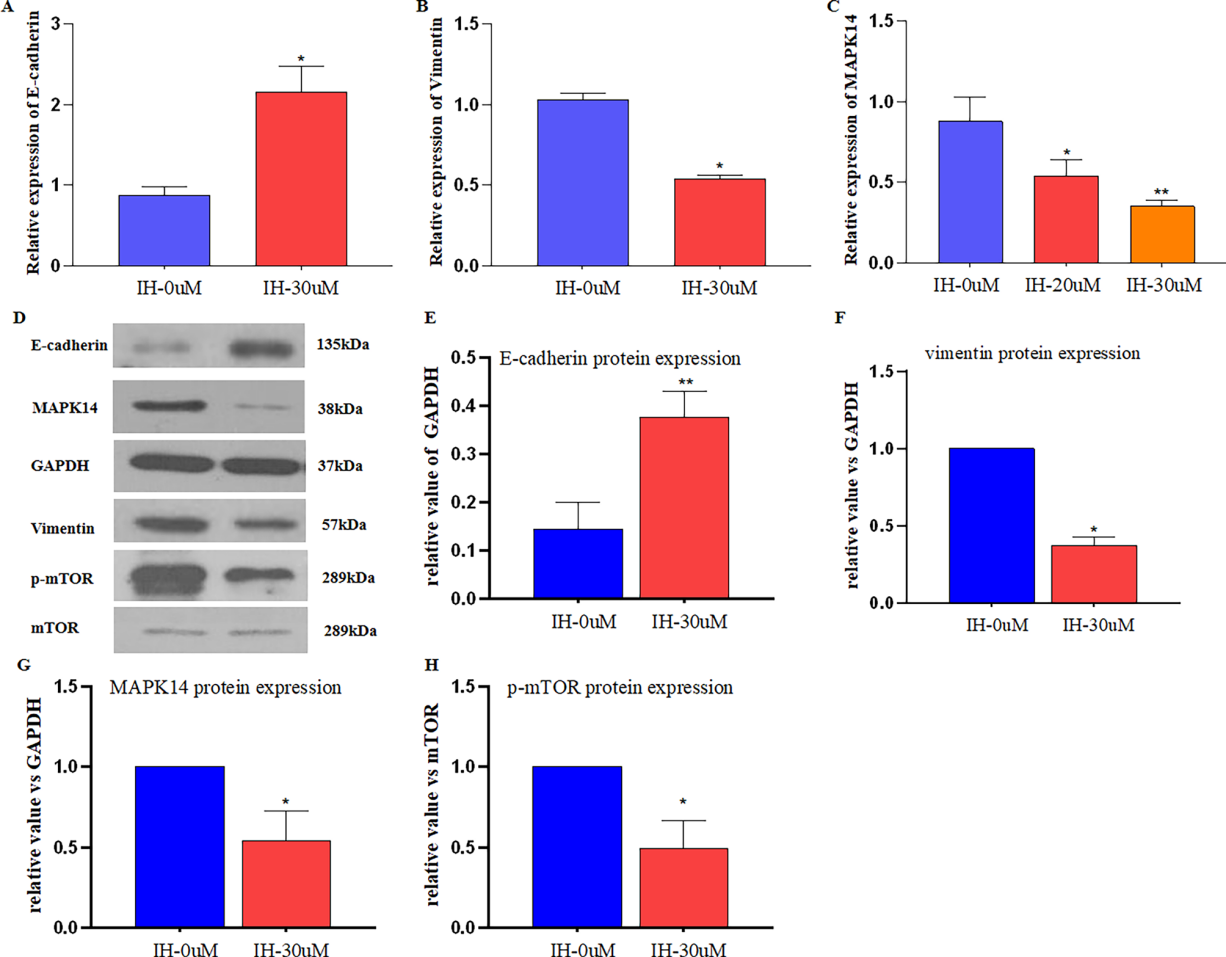
E-cadherin, vimentin, MAPK14 and p-mTOR expression in IH-treated HGC-27 cells. (**A**) The E-cadherin mRNA expression in IH-treated HGC-27 cells detected by qRT-PCR. (**B**) The vimentin mRNA expression in IH-treated HGC-27 cells detected by qRT-PCR. (**C**)The MAPK14 transcription in IH-treated HGC-27 cells detected by qRT-PCR. (**D**) The E-cadherin, vimentin, MAPK14 and p-mTOR protein expression in IH-treated HGC-27 cells detected by western blot. (**E**) E-cadherin bands densitometry quantified using Image J. (**F**) viementin bands densitometry quantified by Image J. (**G**) MAPK14 band densitometry quantified using Image J. (**H**) p-mTOR band densitometry quantified using Image J. Values are mean ± SD, n = 3, *P < 0.05, **P < 0.01 vs. control.

We analyzed the relationship between IH and EMT in HGC-27 cells. qRT-PCR and Western blot analysis showed IH significantly increased E-cadherin (epithelial marker) (Fig. 7A,D,E) and decreased Vimentin (mesenchymal marker) protein and mRNA levels (Fig. 7B,D,F) in HGC-27 cells treated with IH compared with control HGC-27 cells.

### IH regulates the MAPK/mTOR pathway in HGC-27 cells

To detect the effect of IH on MAPK14 expression, we measured the MAPK14 mRNA levels and protein expression in HGC-27 cells treated with or without IH. IH inhibited MAPK14 mRNA expression in a dose-dependent manner (Fig. 7C), and significantly inhibited MAPK14 protein expression (Fig. 7D,G). Study showed that Berberine repressed human gastric cancer cell growth in vitro and in vivo via inhibition of MAPK/mTOR pathway^26^. Therefore, we performed western blot analysis of p-mTOR and mTOR protein expression in HGC-27 cells. Results showed that p-mTOR expression was inhibited in IH treated HGC-27 cells (Fig. 7D,H).

## Discussion

In this study we introduced the relationship between Croci Stigma active ingredients and STAD by their related targets to capture the network of drug to disease genes. The systematic analysis of Croci Stigma and STAD shows that Croci Stigma active ingredients might regulate STAD progression by targeting their core genes MAPK14 and MAPK/mTOR signaling pathway.

Stomach cancer, also called gastric cancer, is the fifth most-common cancer in the twenty-first century. 5-year overall survival rate of advanced Stomach cancer is merely 5%, new therapeutic options thus are urgently required^27^. At present, genomLic analyses have been the major methodology applied for discovering novel biology targets in gastric cancer^28^. In this study, 45 STAD-related genes were screened by DisGeNET platform. And the signaling pathway of these genes enriched in foxo signaling pathway, VEGFA-VEGFR2 pathway, mTOR signaling pathway, and programmed cell death. Otherwise, these STAD-related genes mainly enriched in in protein kinase activity, transmembrane receptor protein kinase activity, protein autophosphorylation, response to growth factor.

Croci Stigma, stigma of Crocus sativus L., is a precious traditional Chinese medicine, which is commonly used to activate blood circulation and to dissipate blood stasis^16^. Crocin, one of the active compounds of Croci Stigma, has been reported to have inhibitory effect against gastric carcinoma and increase gastric cancer cell’s sensitivity to chemotherapy drugs^29,30^. Other active ingredients of Croci Stigma, like Crocetin, also have anti-tumor effect^31^. In the current study, we screened the active ingredients of Croci Stigma by TCMSP database, and 5 active ingredients including n-heptanal, crocetin, isorhamnetin, kaempferol, and quercetin were obtained by OB ≥ 30%, and DL ≥ 0.18. 188 related targets of these 5 active ingredients were obtained and Go enrichment results showed that the 5 active ingredient related genes mainly enriched in apoptotic signaling pathway, regulation of cell adhesion, transcription factor binding, response to oxygen levels, response to extracellular stimulus, reactive oxygen species metabolic process, which are all associated with cancer progression. KEGG pathway showed Croci Stigma active compounds enriched in pathway in cancer, signaling by receptor tyrosine kinase, signaling by nuclear receptors, P53 signaling pathway, ErbB signaling pathway, NF-Kappa B signaling pathway, cell cycle, interleukin-10 signaling, and transcriptional regulation by RNUX2, which are also related to cancer progression. Moreover, 45 STAD-related genes were screened by DisGeNET platform, and the signaling pathway of these genes mainly enriched in foxo signaling pathway, VEGFA-VEGFR2 pathway, mTOR signaling pathway, and programmed cell death as well as protein kinase activity, transmembrane receptor protein kinase activity, protein autophosphorylation, response to growth factor.

Based on above analyses, only 2 core genes (MAPK14 and ERBB3) between active ingredients of Croci Stigma and STAD were obtained. Go enrichment of the two core genes by KOBAS platform showed that both genes enriched in positive regulation of gene expression, signaling transduction, and protein binding. GEPIA2 platform showed the positive correlation between these 2 core genes in STAD patients. Of them, MAPK14 is the related gene of isorhamnetin (IH), and ERBB3 is the related gene of quercetin. TCGA platform showed both MAPK14 and ERBB3 upregulated in STAD patients, but only the effect of MAPK14 expression on STAD patient survival was significant.

MAPK/p38 is an essential component of the MAPK signaling pathway and plays a critical role in the signaling cascades triggered by extra- or intra-cellular stimuli such as inflammatory cytokines or physical stress, resulting in direct activation of transcription factors^32^. Moreover, targeting MAPK/p38 has shown promising therapeutic potential in multiple cancers^33–35^. MAPK14, as one of p38 proteins, was found to be a potential biomarker for advanced gastric cancer as well as a pharmacological target^36,37^. In present work, TCGA database results showed MAPK14 was significantly upregulated in gastric cancer samples compared with normal samples, especially in advanced stages (stage 3 and 4). But there was no significant difference in MAPK14 expression between male and female STAD patients, and the increase of MAPK14 expression was not associated with H. pylori infection. Most important of all, MAPK14 expression was significantly associated with patient survival with low/medium MAPK14 expression having a better survival probability.

MAPK14 is the related gene of isorhamnetin (IH), and IH, one of the active components of Croci Stigma, has antioxidant, organ protection, anti-inflammatory, and antitumor activity^38–40^. Molecular docking results showed that IH had a significant role in regulation of MAPK14 protein expression. Consistently, our experimental results further verified that IH inhibited HGC-27 cell proliferation, migration, and colony formation, and HGC-27 cell apoptosis by inhibiting MAPK14 expression. Moreover, Berberine repressed human gastric cancer cell growth in vitro and in vivo via inhibition of MAPK/mTOR pathway^25^. In our study, IH also inhibited the expression of p-mTOR in HGC-27 cells. Together, these findings revealed that IH, one of active ingredient of Croci Stigma, has therapeutic potential for the treatment of STAD by inhibiting MAPK14 expression and mTOR signaling.

## Conclusion

In summary, network pharmacology showed that the active components of Croci Stigma may act on multiple targets, and have the effect to treat STAD by regulating several pathways, such as VEGF pathway, Fc epsilon RI signaling pathway, RIG-I-like receptor signaling pathway, ErbB signaling pathway, Calcium signaling pathway, and PI3K-AKT signaling pathway. Data analysis from TCGA platform showed MAPK14 expression was upregulated in STAD patients, which was associated with STAD patients’ survival. Our molecular docking and experiment results further showed that IH decreased MAPK14 expression, inhibited HGC-27 cell proliferation and migration, promoted HGC-27 cell apoptosis, induced cell cycle arrest, having the therapeutic potential for the treatment of STAD.

### Supplementary Information

Supplementary Figure S1.

Supplementary Figure S2.

Supplementary Figure S3.

Supplementary Figure S4.

Supplementary Information 5.

Supplementary Information 6.

Supplementary Information 7.

Supplementary Information 8.

Supplementary Information 9.

Supplementary Information 10.

Supplementary Information 11.

Supplementary Information 12.

Supplementary Information 13.

Supplementary Information 14.

Supplementary Information 15.

Supplementary Information 16.

Supplementary Tables.

## Data Availability

Croci Stigma active ingredients were screened from TCMSP database (https://old.tcmsp-e.com/tcmsp.php), and DisGeNET platform (DisGeNET—a database of gene-disease associations) were used to screen STAD targets.
