# Supplementary figures and images for: Active ingredients Isorhamnetin of Croci Srigma inhibit stomach adenocarcinomas progression by MAPK/mTOR signaling pathway
Source: Sci Rep. 2023 Aug 3;13:12607. doi: 10.1038/s41598-023-39627-z (PMC10400561; doi:10.1038/s41598-023-39627-z)

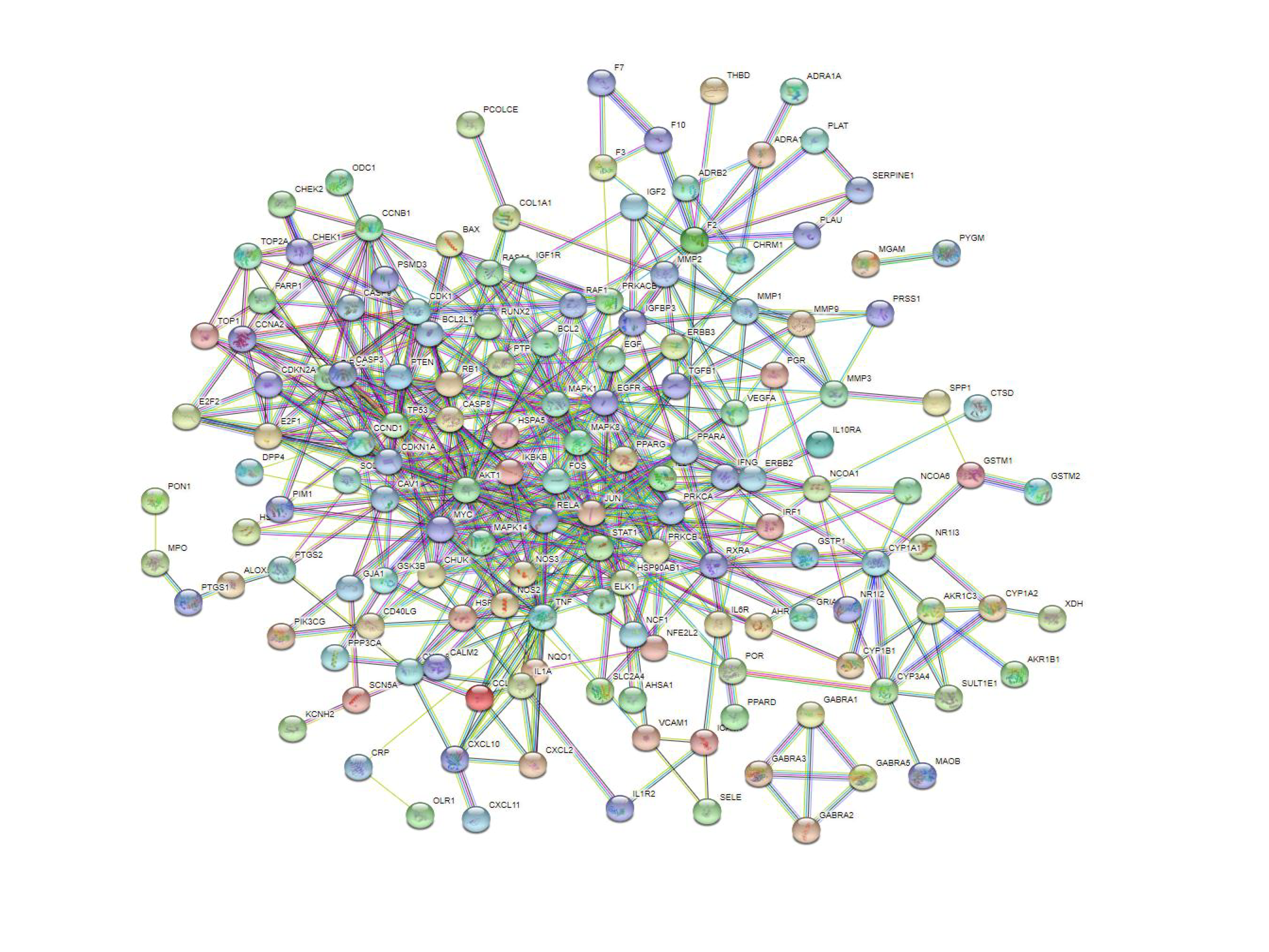

Supplement: Supplementary file 1 — Supplementary Figure S1. [file 41598_2023_39627_MOESM1_ESM.tif]

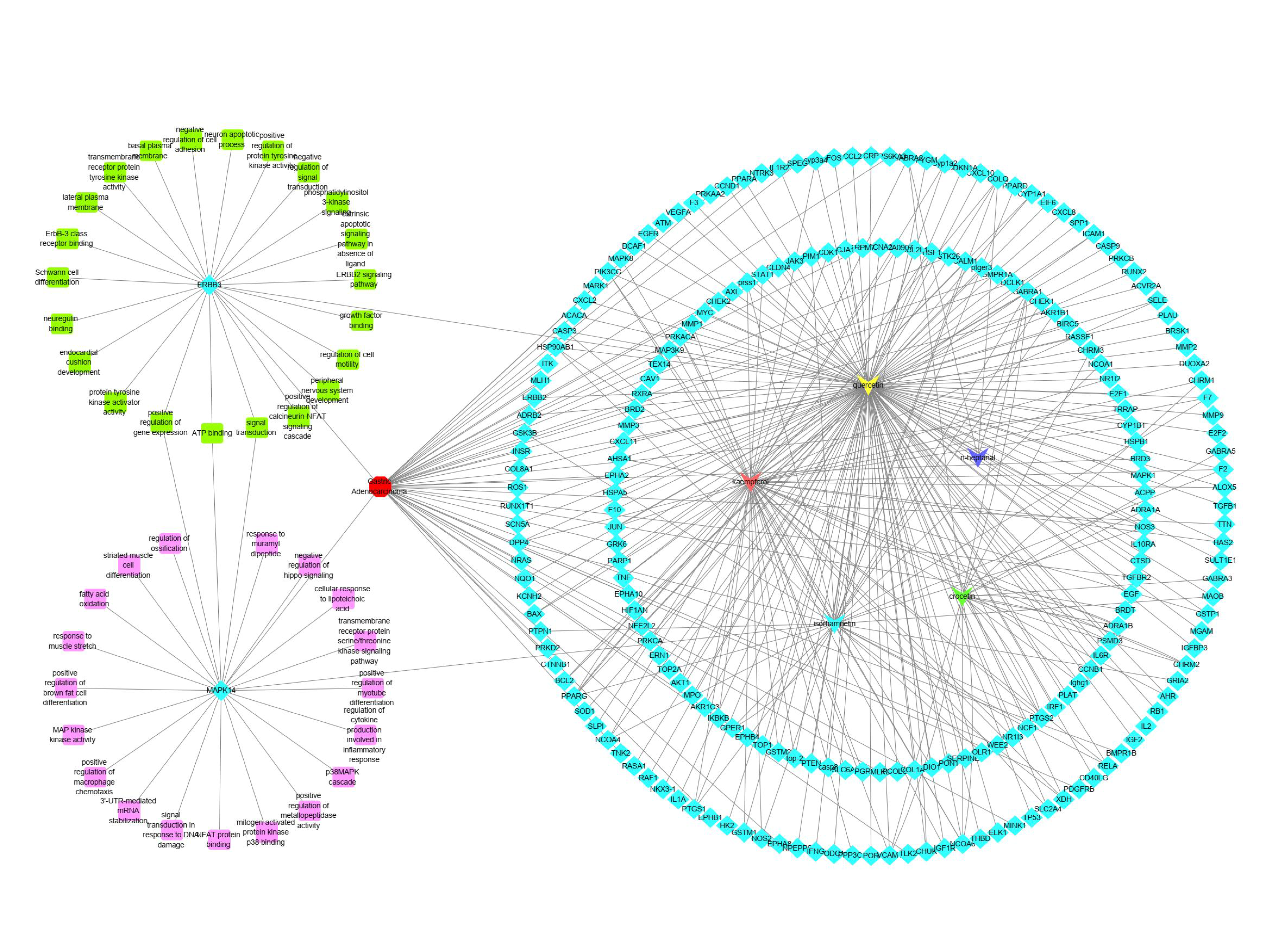

Supplement: Supplementary file 2 — Supplementary Figure S2. [file 41598_2023_39627_MOESM2_ESM.tif]

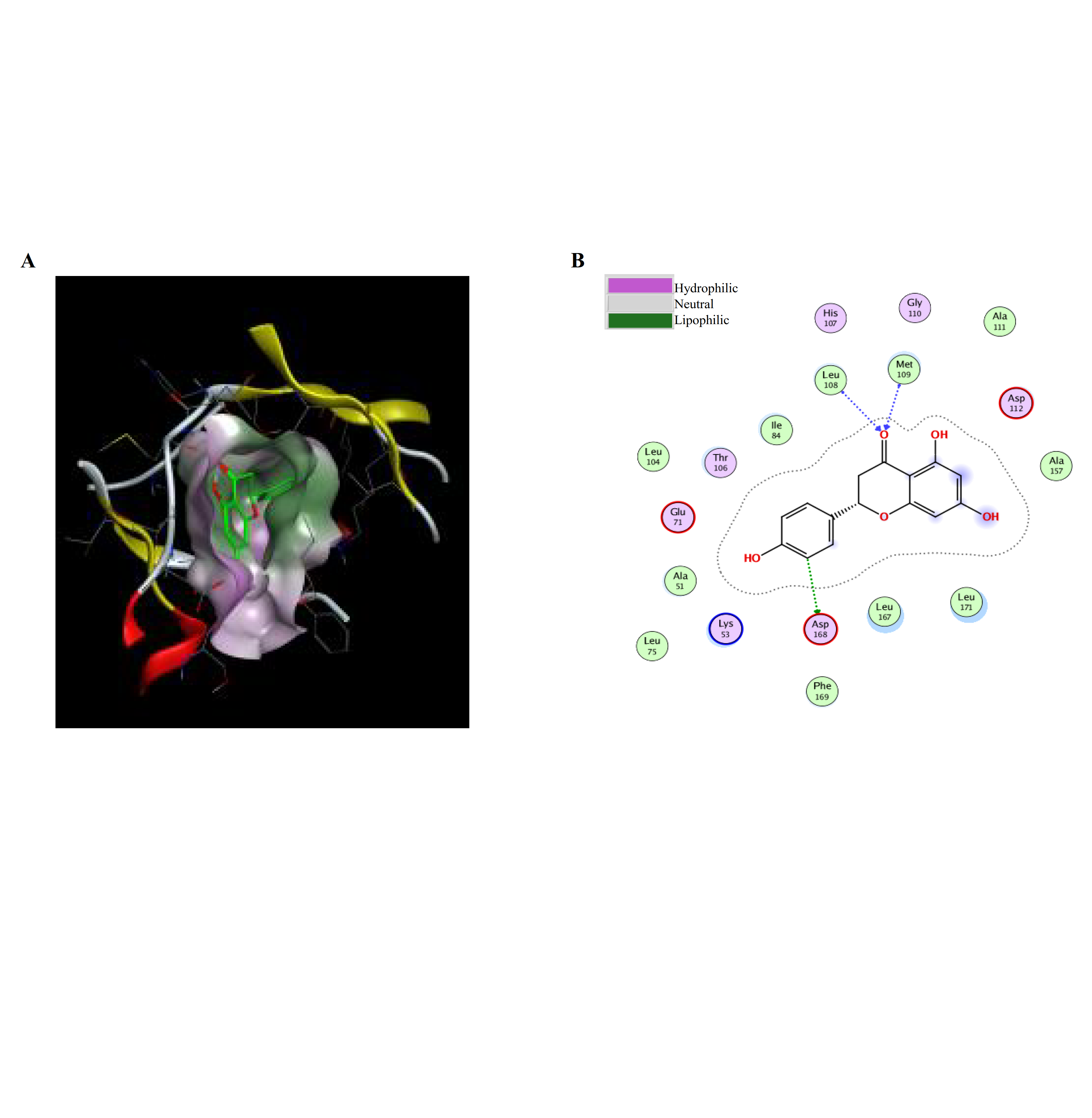

Supplement: Supplementary file 3 — Supplementary Figure S3. [file 41598_2023_39627_MOESM3_ESM.tif]

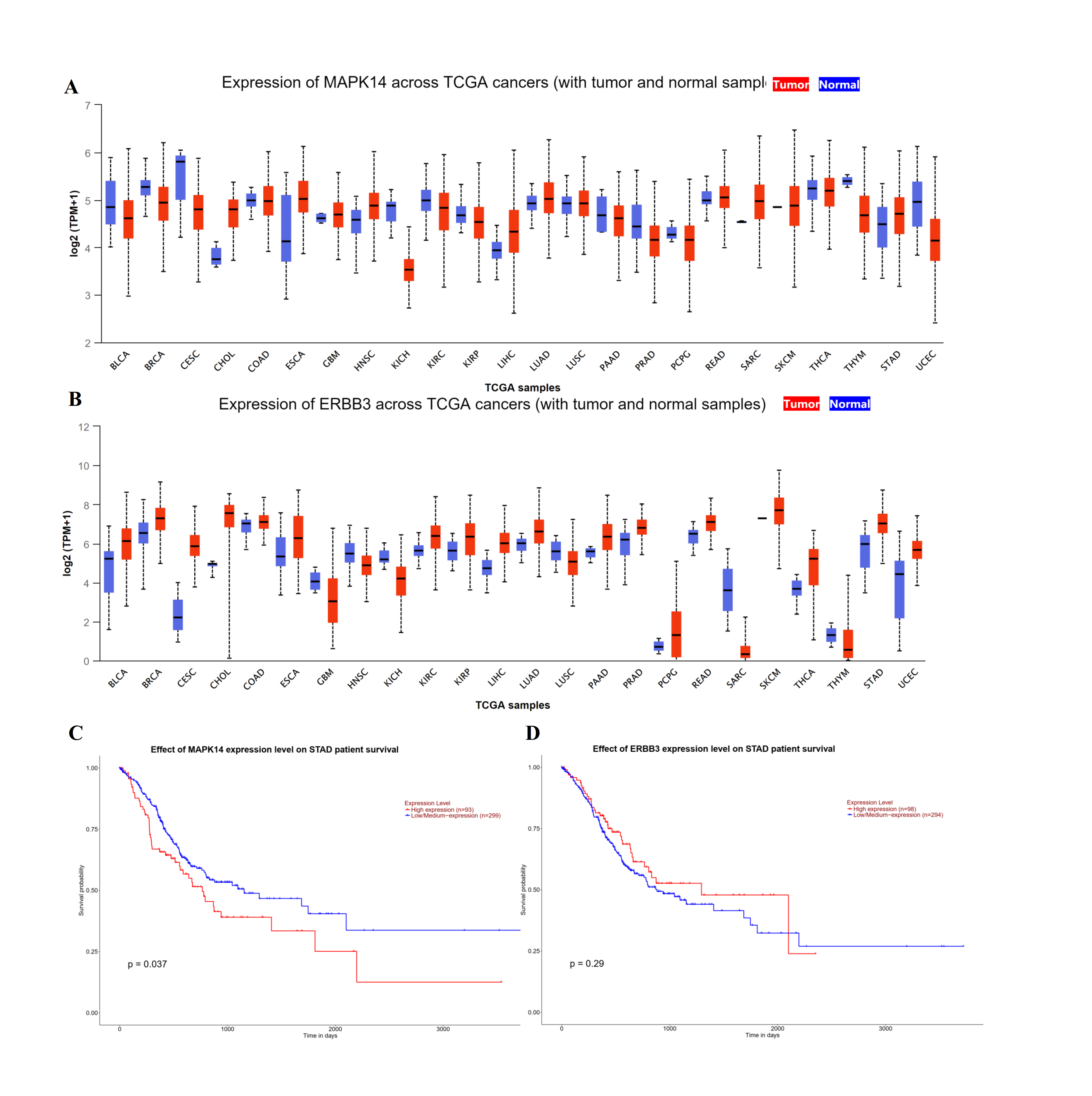

Supplement: Supplementary file 4 — Supplementary Figure S4. [file 41598_2023_39627_MOESM4_ESM.tif]

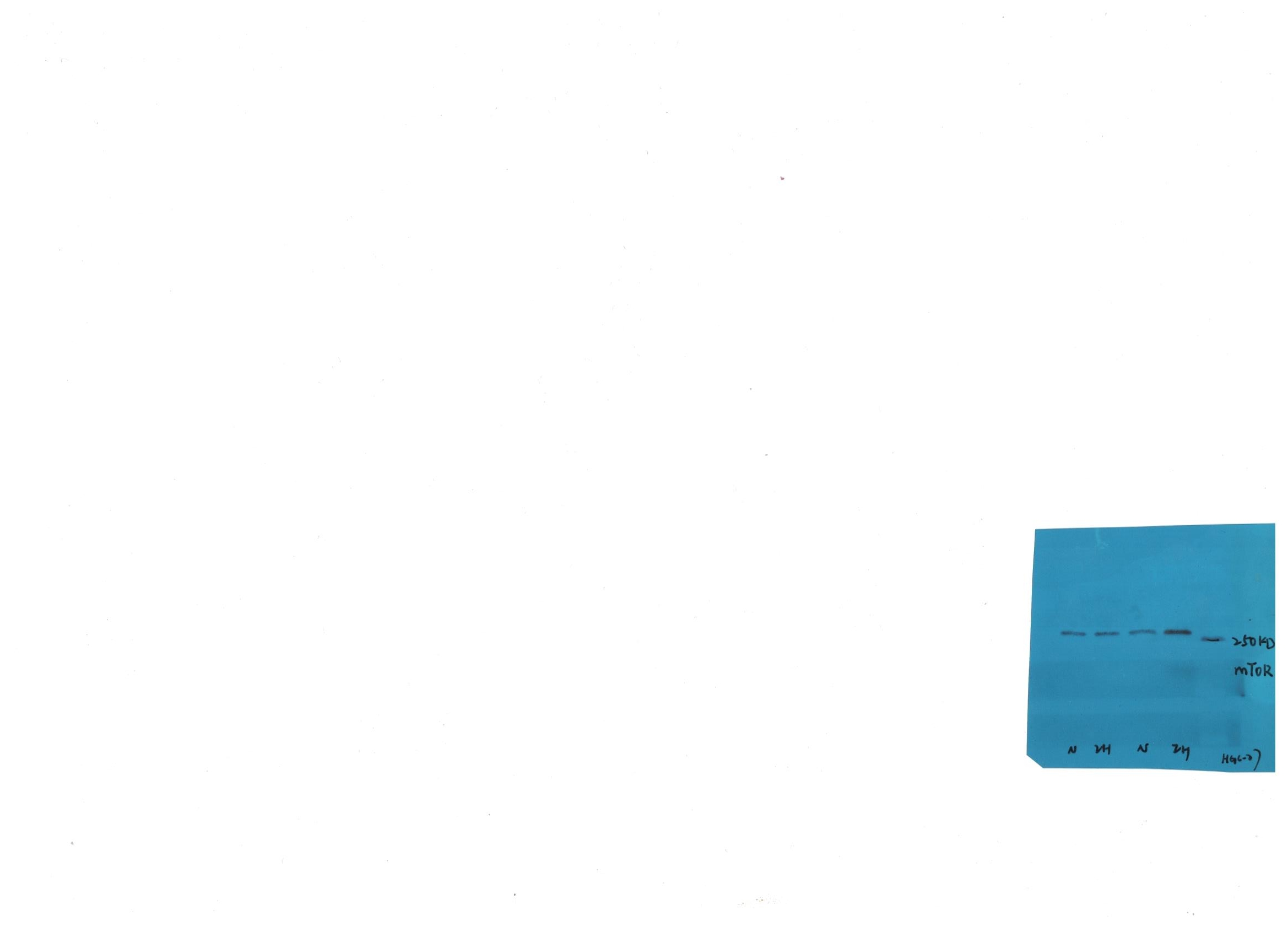

Supplement: Supplementary file 5 — Supplementary Information 5. [file 41598_2023_39627_MOESM5_ESM.jpg]

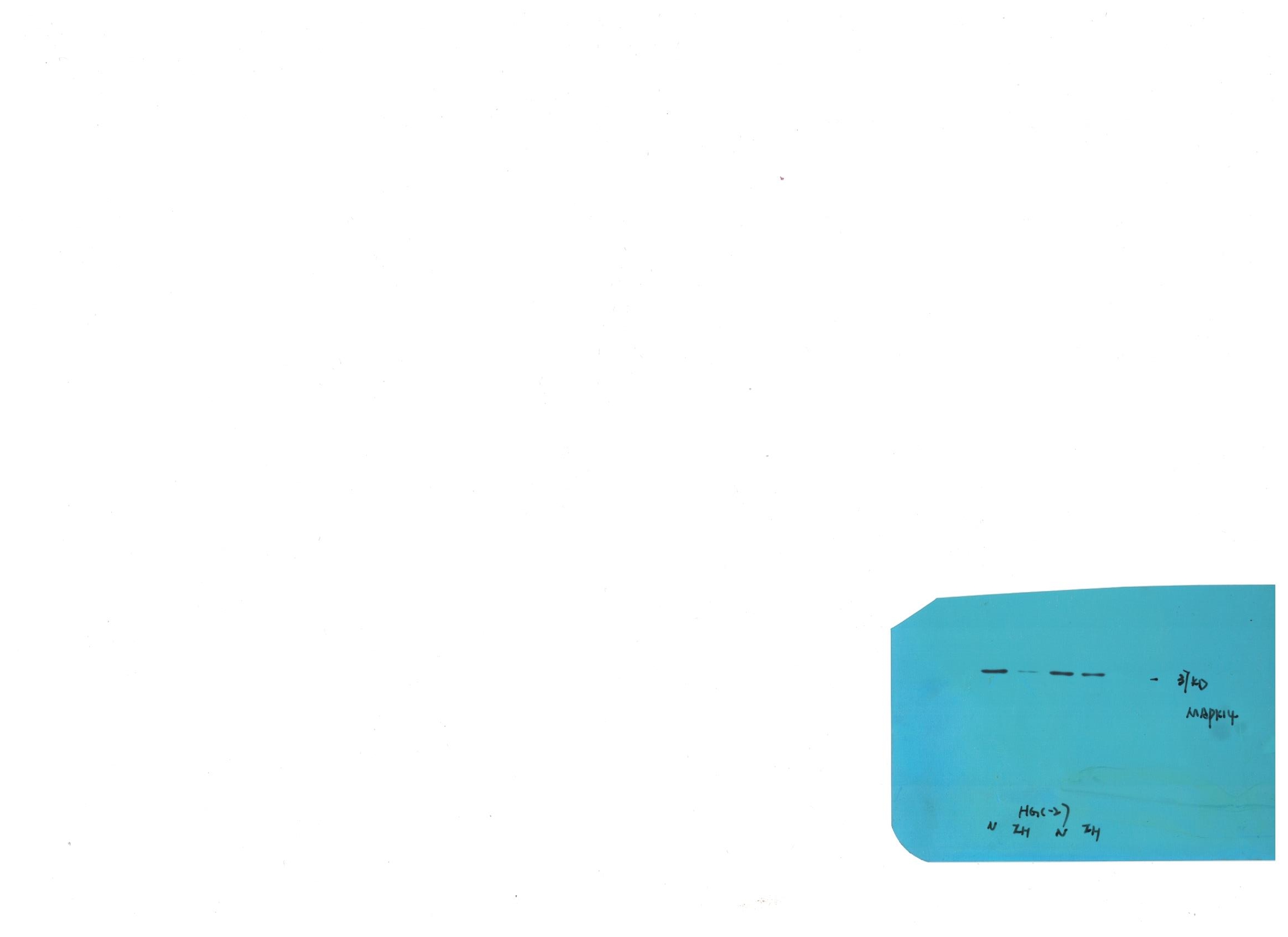

Supplement: Supplementary file 6 — Supplementary Information 6. [file 41598_2023_39627_MOESM6_ESM.jpg]

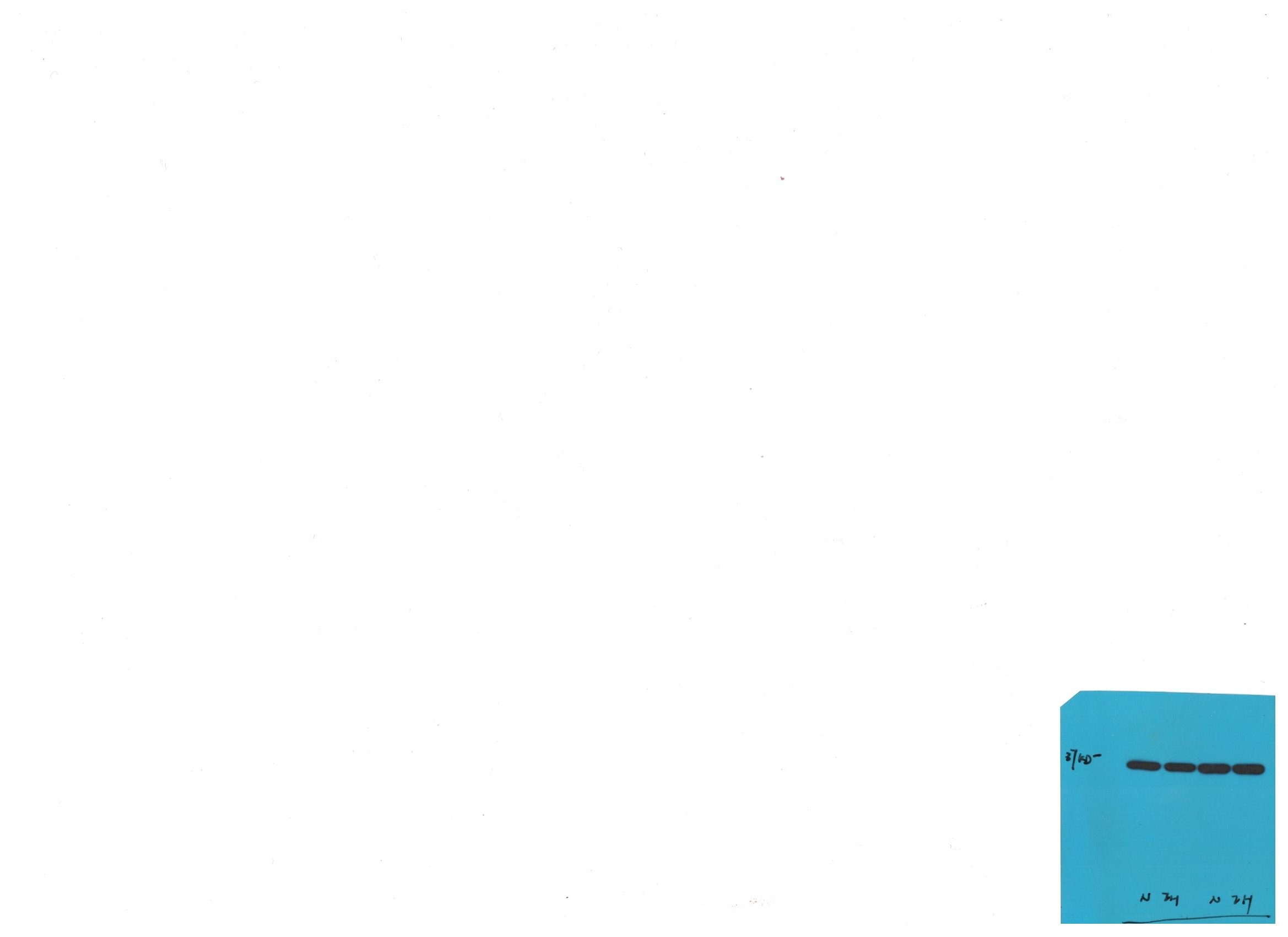

Supplement: Supplementary file 7 — Supplementary Information 7. [file 41598_2023_39627_MOESM7_ESM.jpg]

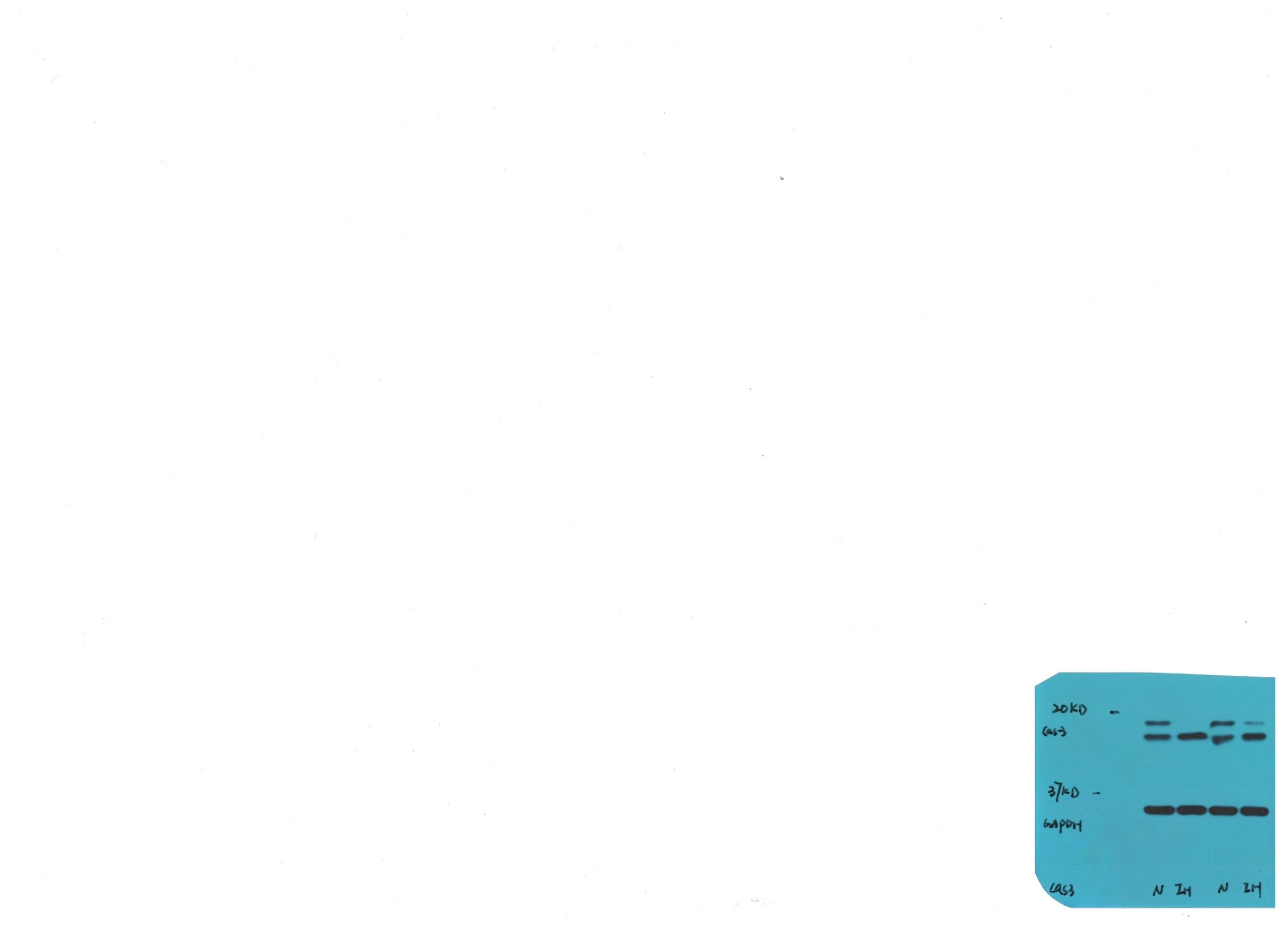

Supplement: Supplementary file 8 — Supplementary Information 8. [file 41598_2023_39627_MOESM8_ESM.jpg]

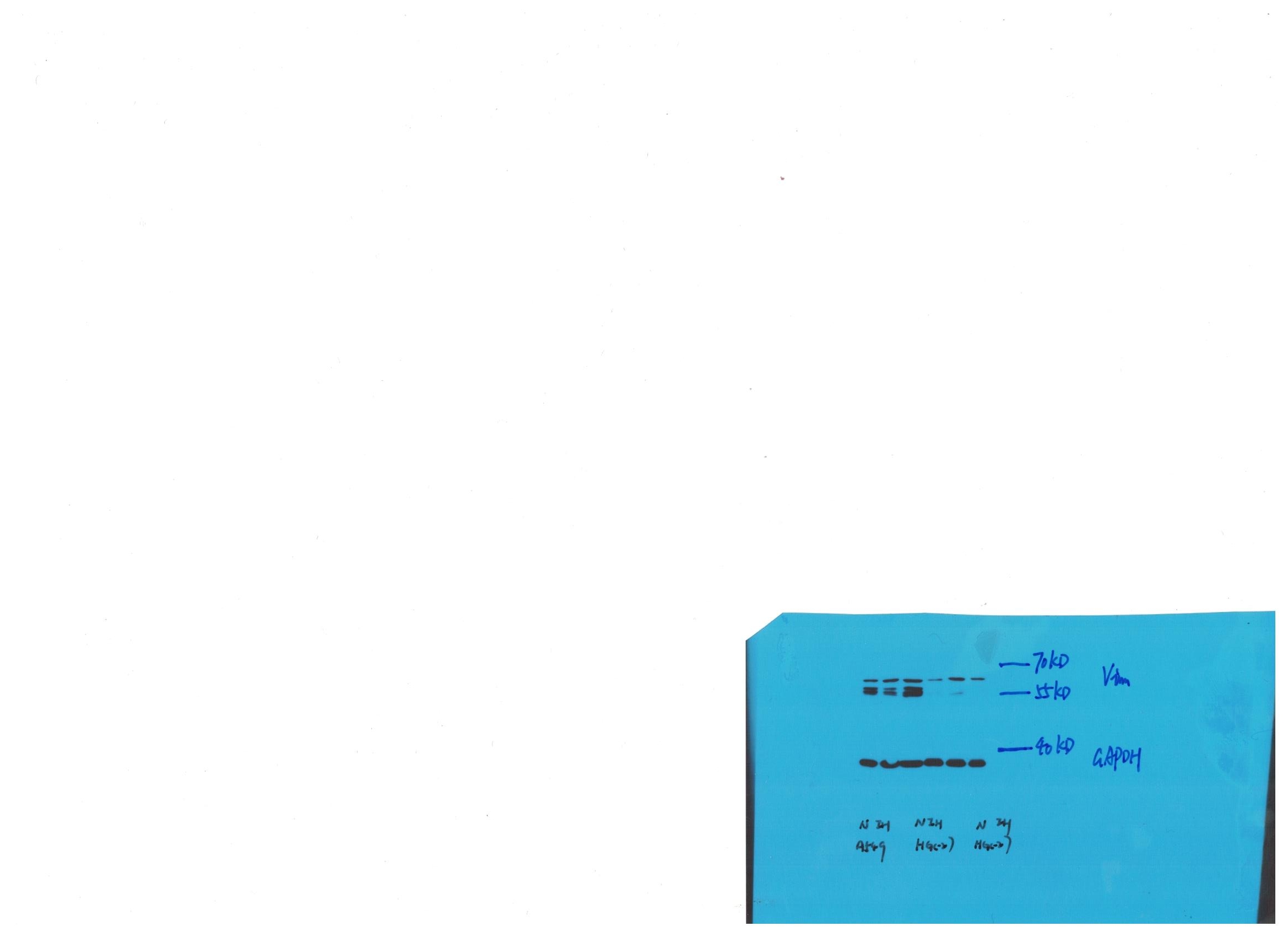

Supplement: Supplementary file 9 — Supplementary Information 9. [file 41598_2023_39627_MOESM9_ESM.jpg]

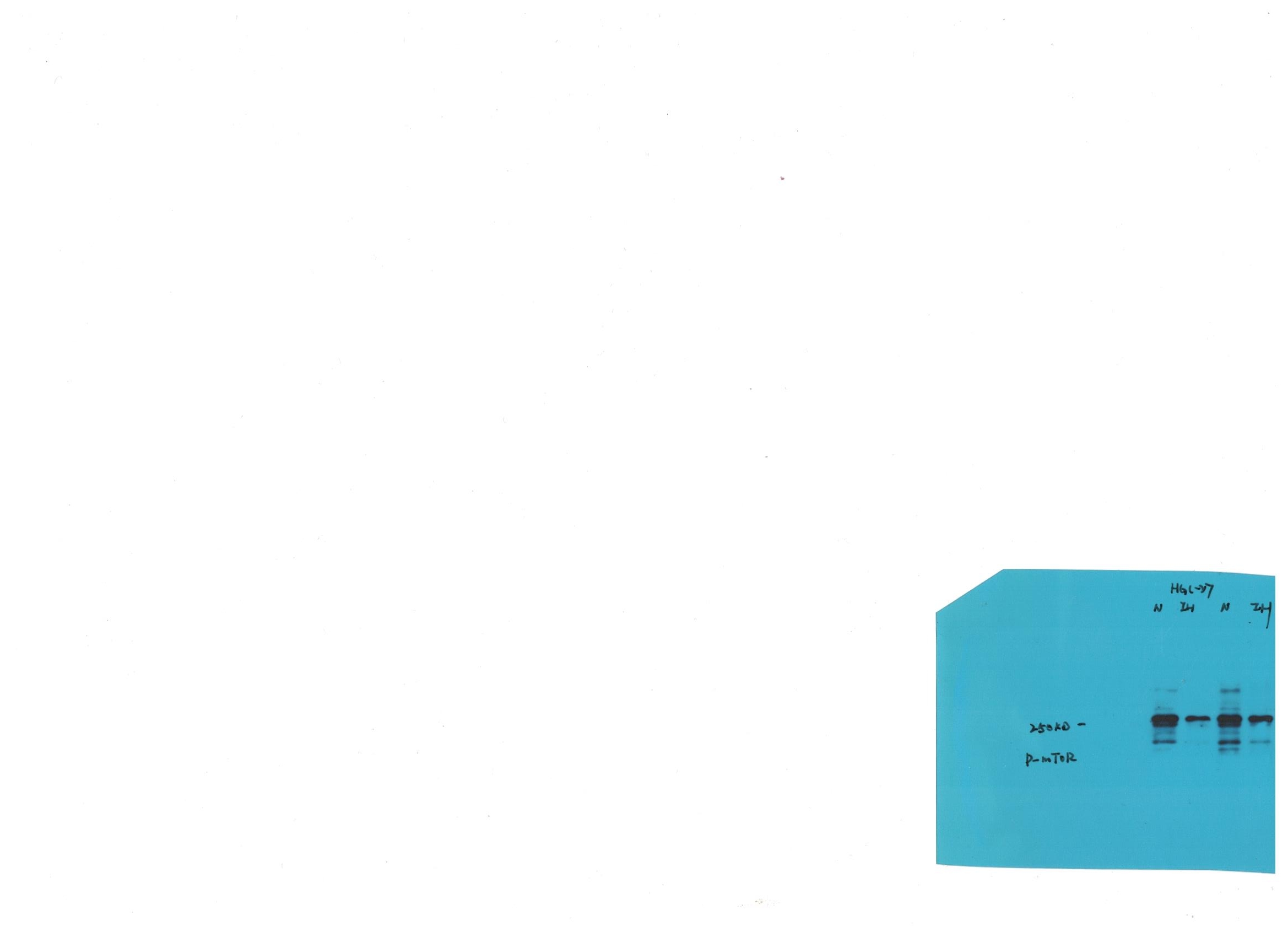

Supplement: Supplementary file 10 — Supplementary Information 10. [file 41598_2023_39627_MOESM10_ESM.jpg]

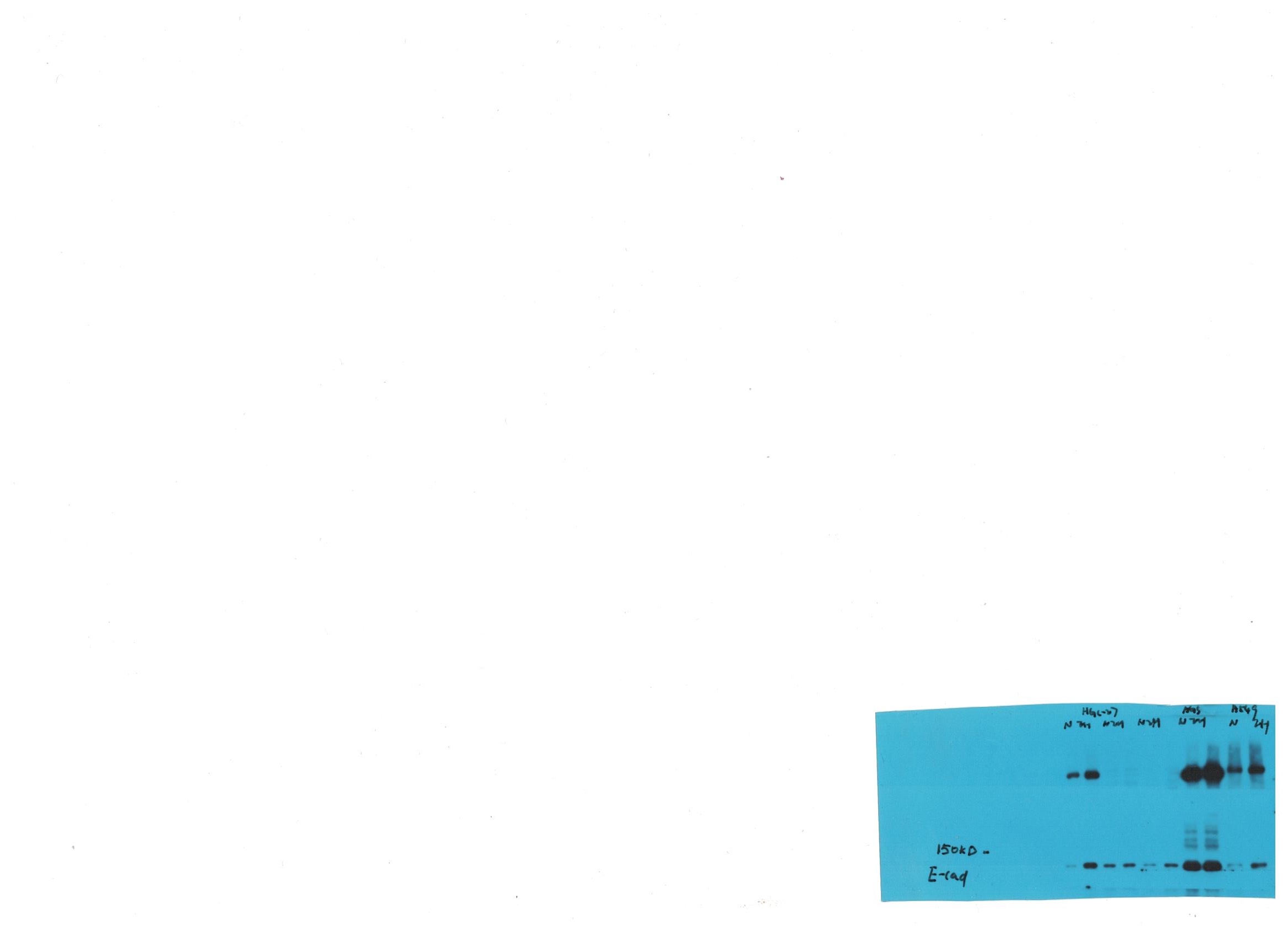

Supplement: Supplementary file 11 — Supplementary Information 11. [file 41598_2023_39627_MOESM11_ESM.jpg]

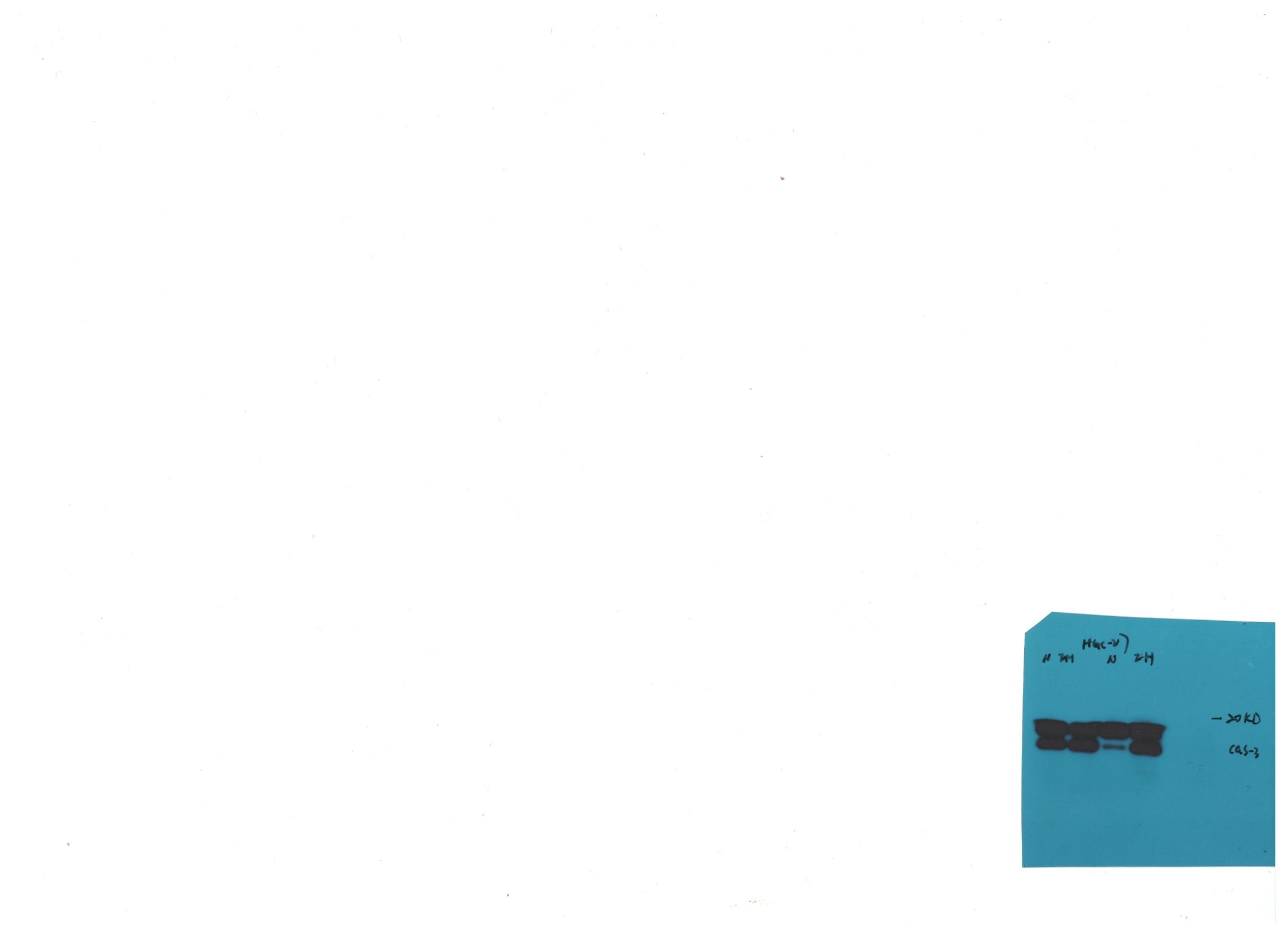

Supplement: Supplementary file 12 — Supplementary Information 12. [file 41598_2023_39627_MOESM12_ESM.jpg]

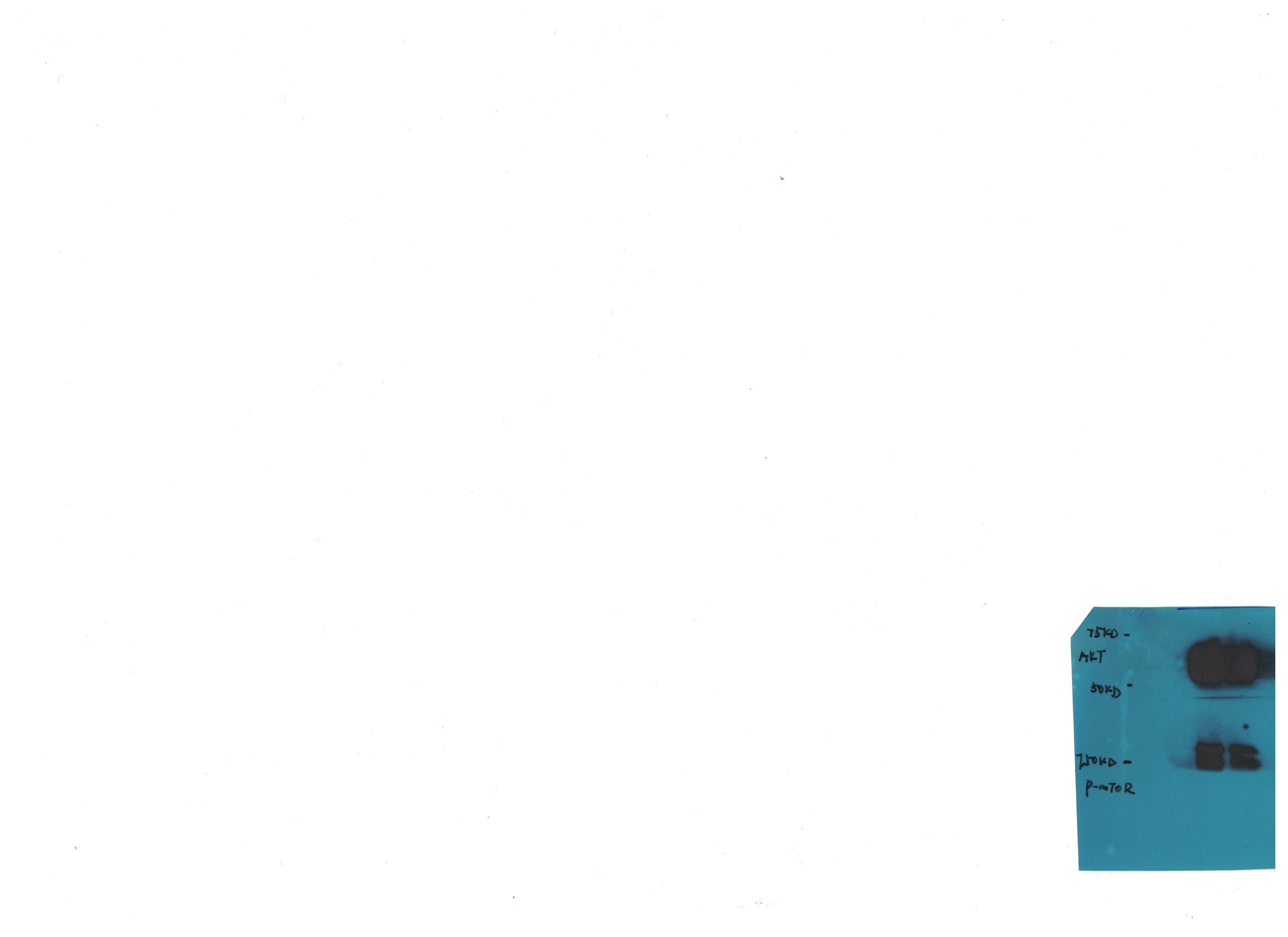

Supplement: Supplementary file 13 — Supplementary Information 13. [file 41598_2023_39627_MOESM13_ESM.jpg]

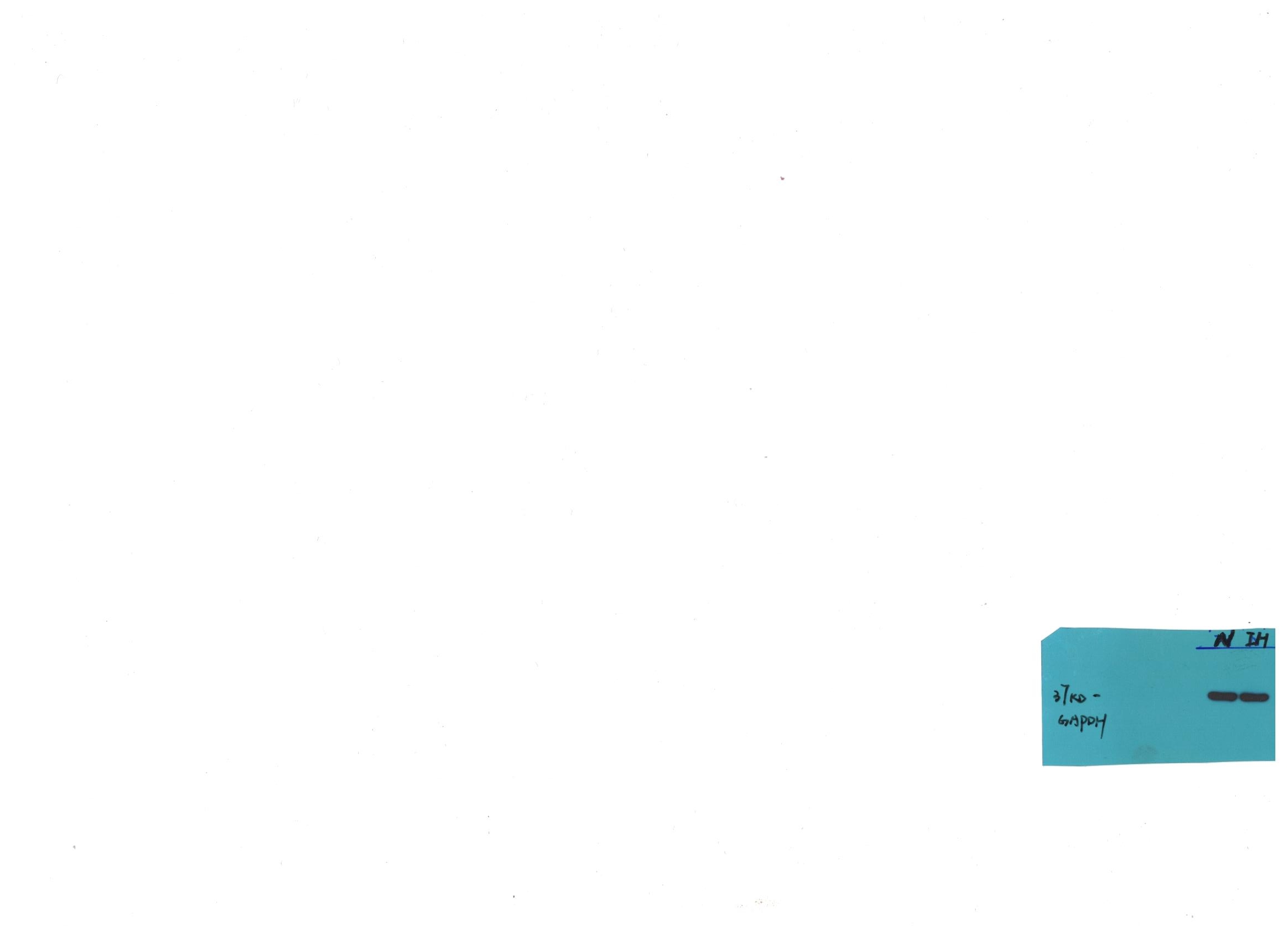

Supplement: Supplementary file 14 — Supplementary Information 14. [file 41598_2023_39627_MOESM14_ESM.jpg]

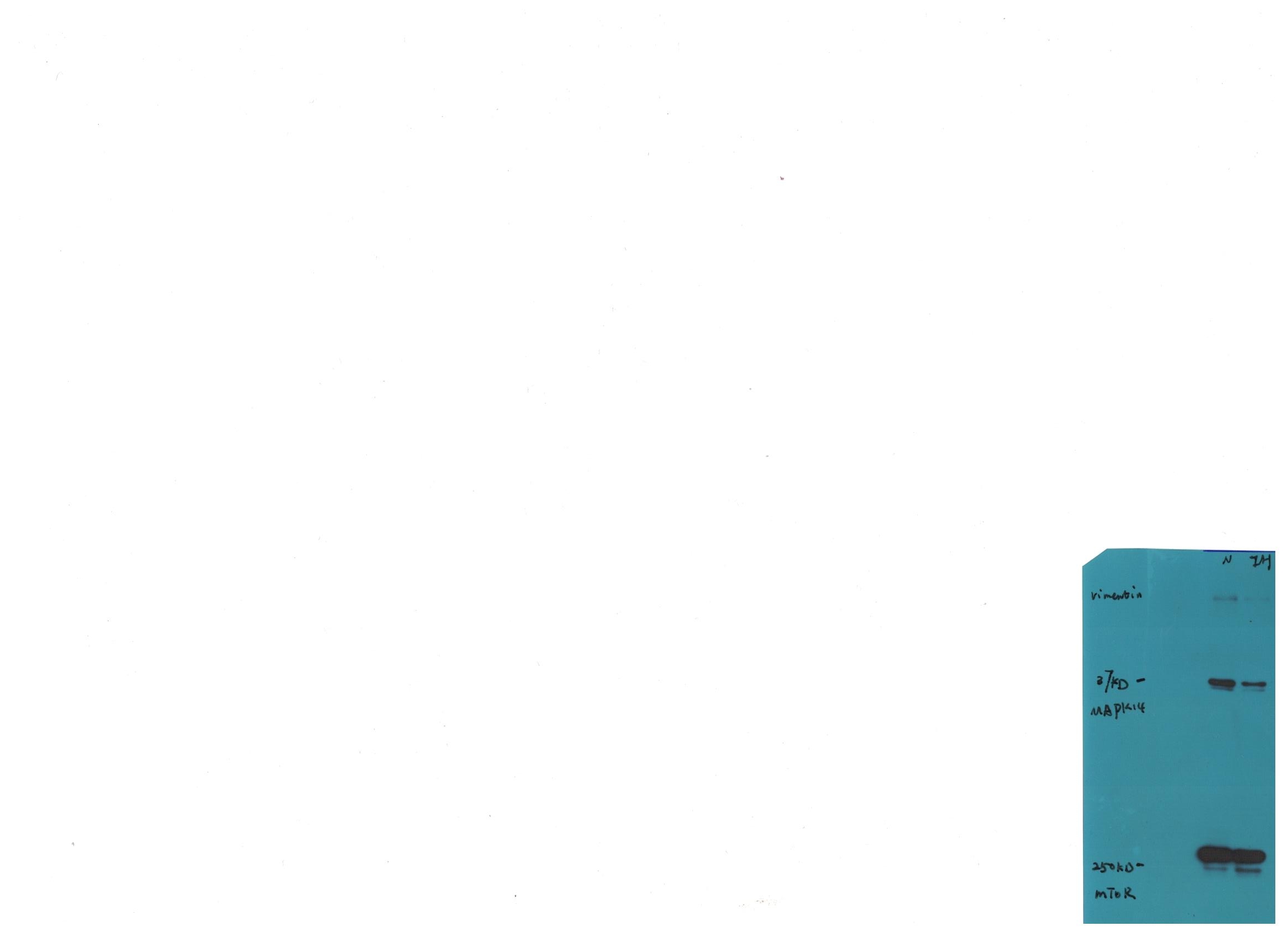

Supplement: Supplementary file 15 — Supplementary Information 15. [file 41598_2023_39627_MOESM15_ESM.jpg]

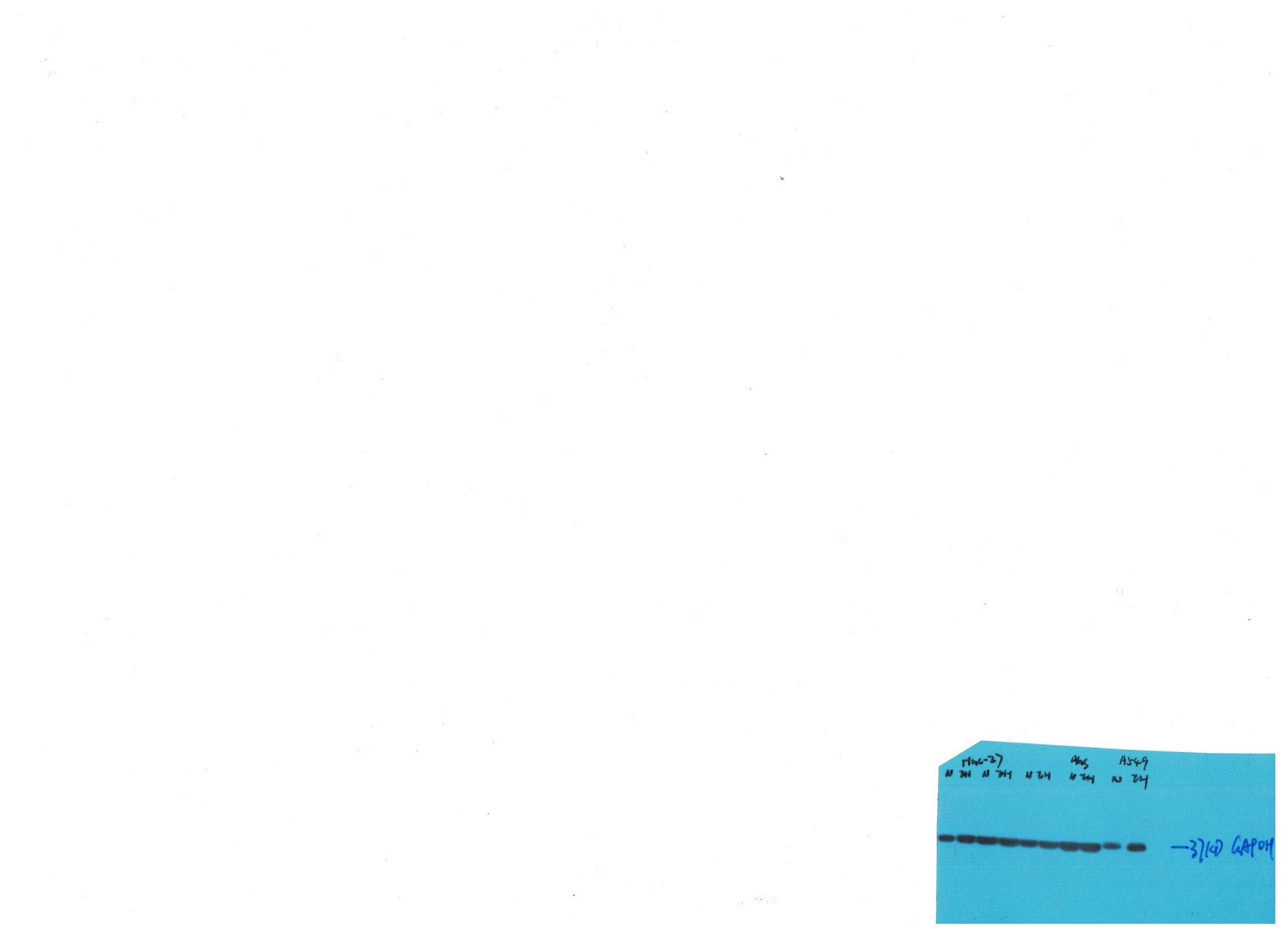

Supplement: Supplementary file 16 — Supplementary Information 16. [file 41598_2023_39627_MOESM16_ESM.jpg]
