# Supplementary material for: Active ingredients Isorhamnetin of Croci Srigma inhibit stomach adenocarcinomas progression by MAPK/mTOR signaling pathway
Source: Sci Rep. 2023 Aug 3;13:12607. doi: 10.1038/s41598-023-39627-z (PMC10400561; doi:10.1038/s41598-023-39627-z)
Supplement: Supplementary file 17 — Supplementary Tables. [file 41598_2023_39627_MOESM17_ESM.docx]

Supplementary Table 1. Active ingredients of Croci Stigma from TCMSP platform

| Molecule name | [OB (%)](https://old.tcmsp-e.com/tcmspsearch.php?qr=Croci%20Stigma&qsr=herb_en_name&token=37e7ba346324682a73bb9b3b160faa6b) | [DL](https://old.tcmsp-e.com/tcmspsearch.php?qr=Croci%20Stigma&qsr=herb_en_name&token=37e7ba346324682a73bb9b3b160faa6b) | Structure |
| --- | --- | --- | --- |
| n-heptanal | 79.74 | 0.59 | 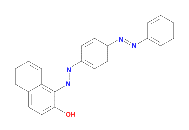 |
| Crocetin | 35.30 | 0.26 | 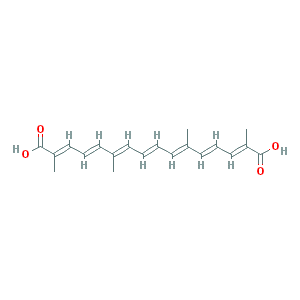 |
| Isorhamnetin | 49.60 | 0.31 | 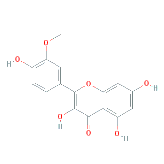 |
| Kaempferol | 41.88 | 0.24 | 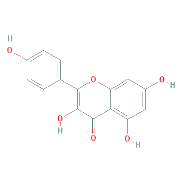 |
| Quercetin | 46.43 | 0.28 | 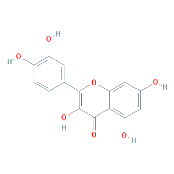 |

Supplementary Table2. The interaction of Croci Stigma active ingredients targets protein

| Protein 1 | Protein 2 | Interaction Score |
| --- | --- | --- |
| MAPK14 | AKT1 | 0.956 |
| MAPK14 | CASP3 | 0.977 |
| MAPK14 | CD40LG | 0.925 |
| MAPK14 | CXCL8 | 0.953 |
| MAPK14 | ELK1 | 0.923 |
| MAPK14 | FOS | 0.966 |
| MAPK14 | HSPB1 | 0.985 |
| MAPK14 | IL2 | 0.938 |
| MAPK14 | JUN | 0.989 |
| MAPK14 | MAPK1 | 0.977 |
| MAPK14 | MYC | 0.954 |
| MAPK14 | NCF1 | 0.956 |
| MAPK14 | RB1 | 0.986 |
| MAPK14 | RELA | 0.955 |
| MAPK14 | STAT1 | 0.963 |
| MAPK14 | TNF | 0.975 |
| MAPK14 | TP53 | 0.995 |
| MAPK14 | VEGFA | 0.951 |
| ERBB3 | AKT1 | 0.988 |
| ERBB3 | EGF | 0.999 |
| ERBB3 | EGFR | 0.997 |
| ERBB3 | ERBB2 | 0.992 |
| ERBB3 | MAPK8 | 0.926 |

Supplementary Table3. Subtype descriptions and pathologic N descriptions

| Subtype/Pathologic N | Descriptions |
| --- | --- |
| AdenoNOS | Adenocarcinoma NOS |
| AdenoDiffuse | Adenocarcinoma Diffuse |
| AdenoSignetRing | Adenocarcinoma SignetRing |
| IntAdenoNOS | IntestinalAdenocarcinoma NOS |
| IntAdenoTubular | IntestinalAdenocarcinoma Tubular |
| IntAdenoMucinous | IntestinalAdenocarcinoma Mucinous |
| IntAdenoPapillary | IntestinalAdenocarcinoma Papillary |
| Grade1 | well differentiated (low grade) |
| Grade2 | moderately differentiated (intermediate grade) |
| Grade3 | poorly differentiated (high grade) |
| Grade4 | Undifferentiated (high grade) |
| N0 | No regional lymph node metastasis |
| N1 | Metastases in 1 to 3 axillary lymph nodes |
| N2 | Metastases in 4 to 9 axillary lymph nodes |
| N3 | Metastases in 10 or more axillary lymph nodes |
